# Mode Crystallography Analysis through the Structural Phase Transition and Magnetic Critical Behavior of the Lacunar Spinel GaMo_4_Se_8_

**DOI:** 10.1021/acs.chemmater.1c01448

**Published:** 2021-07-06

**Authors:** Kieran Routledge, Praveen Vir, Nicholas Cook, Philip A. E. Murgatroyd, Sheikh J. Ahmed, Stanislav N. Savvin, John B. Claridge, Jonathan Alaria

**Affiliations:** † Department of Physics, University of Liverpool, Oxford Street, Liverpool L69 7ZE, U.K.; ‡ Diffraction Group, Institut Laue-Langevin, 71 Avenue des Martyrs, Grenoble 38000, France; § Department of Chemistry, University of Liverpool, Crown Street, Liverpool L69 7ZD, U.K.

## Abstract

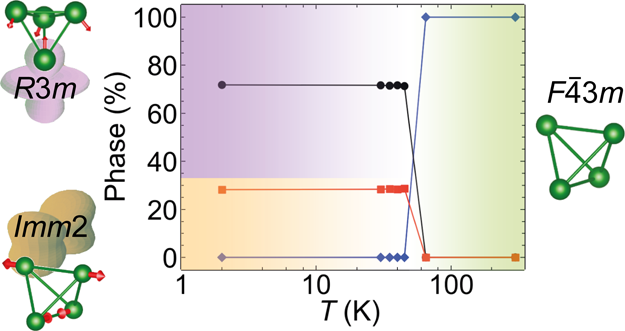

In the lacunar spinels, with the formula AB_4_X_8_, transition-metal ions form tightly bound B_4_ clusters resulting in exotic physical properties such as the stabilization of Néel-type skyrmion lattices, which hold great promise for energy-efficient switching devices. These properties are governed by the symmetry of these compounds with distortion of the parent noncentrosymmetric *F*4̅3*m* space group to the polar *R*3*m*, with recent observation of a coexisting *Imm*2 low-temperature phase. In this study, through powder neutron diffraction, we further confirm that a metastable *Imm*2 coexists with the *R*3*m* phase in GaMo_4_Se_8_ and we present its structure. By applying the mode crystallography approach to the distortions together with anisotropic microstrain broadening analysis, we postulate that the formation origin of the minority *Imm*2 phase stems from the high compressive stress observed in the *R*3*m* phase. Bond valence sum analysis also suggests a change in electronic configuration in the transition to *Imm*2 which could have implications on the electrical properties of the compound. We further establish the nature of the magnetic phase transition using critical exponent analysis obtained from single-crystal magnetization measurements which shows a mixture of tricritical mean-field and 3D Heisenberg behavior [β = 0.22(4), γ = 1.19(1), and δ = 6.42(1)]. Magnetoentropic mapping performed on a single crystal reveals the signature of a positive entropy region near the magnetic phase transition which corresponds to the skyrmion phase field observed in a polycrystalline sample.

## Introduction

The class of compounds known as lacunar spinels, with the formula AB_4_X_8_ (A = Ga and Ge; B = V, Mo, Nb, and Ta; and X = S and Se), provides a diverse chemistry allowing the study of fundamental mechanisms and physical properties, which could be used in energy-efficient switching devices. Examples of the properties that have been observed in these narrow gap Mott insulators^1^ include multiferroicity (in GaV_4_S_8_, GeV_4_S_8_, and GaMo_4_S_8_),^2−6^ resistive switching abilities with promising use in resistive random access memories (in GaTa4Se_8–*y*_Te_*y*_, GaV_4_S_8_, and GaMo_4_S_8_),^7−10^ large negative magnetoresistance (in GaTi_3_VS_8_ and GaV_4_S_8_),^11,12^ and even pressure-induced superconductivity (in GaNb_4_S_8_, GaNb_4_Se_8_, and GaTa_4_Se_8_).^13,14^ In magnetically ordered compounds such as GaV_4_S_8_ and GaV_4_Se_8_, which present a structural phase transition to a polar structure, Néel-type skyrmion lattices have been observed.^2,3,15−17^ GaMo_4_S_8_ and GaMo_4_Se_8_ also exhibit this polar structure and have had multiple metamagnetic states including possible skyrmion lattices observed in them.^18,19^ Skyrmions are topologically stable vortex-like spin structures that could be used in future memory devices,^20−22^ and the different chemistries available in the lacunar spinels offer an opportunity to further improve our fundamental understanding of these states.

**Figure 1:**
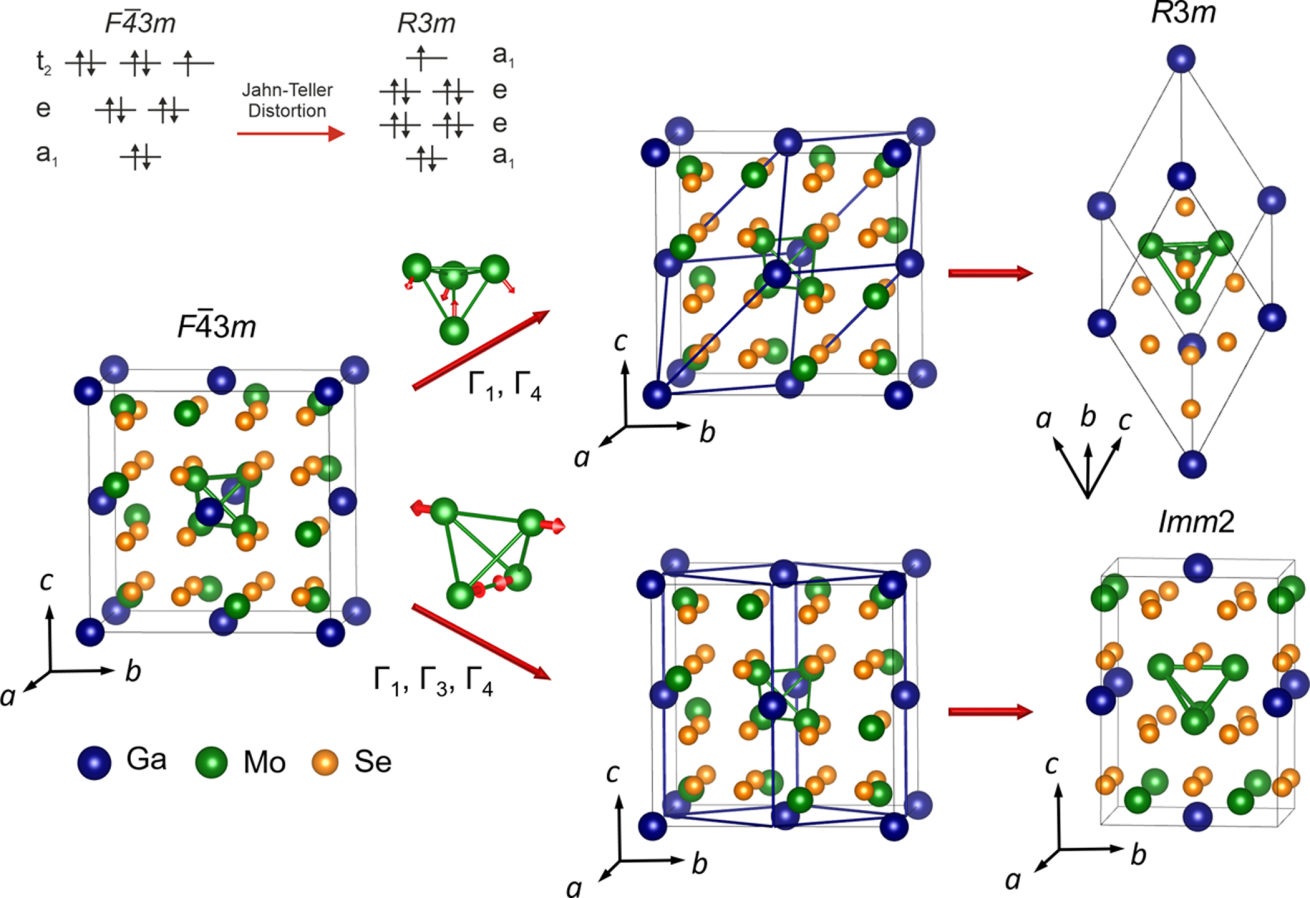
Schematic of structural transitions in GaMo_4_Se_8_ induced by Jahn–Teller distortions. The Mo_4_ tetrahedron in the unit cells is shown with green lines and the two low-temperature structures are displayed within pseudo-cubic cells (indicated by blue solid lines) and on their own. Γ_1_, Γ_3_, and Γ_4_ are the distortion modes. The molecular orbital diagrams are of a Mo_4_Se_4_ heterocubane unit in GaMo_4_Se_8_ through the cubic to rhombohedral transition.

All of these intriguing properties are due to the unique nature of the structure of lacunar spinels, which crystallize in the noncentrosymmetric cubic space group *F*4̅3*m* at room temperature. The cubic structure in the case of GaMo_4_Se_8_ is shown on the left of Figure 1. Unlike a regular AB_2_X_4_ spinel, they have vacancies on every other A site, resulting in the transition-metal cations on B sites to be brought close together into B_4_ clusters in a tetrahedron formation and can therefore be described as a breathing-type spinel. The Mo_4_ tetrahedra are highlighted with green bonds in Figure 1. The structure can also be considered as an NaCl-like lattice of (B_4_X_4_)^*n*+^ heterocubane units (within which are the B_4_ clusters) alternated with (AX_4_)^*n*−^ tetrahedra.

In GaMo_4_S_8_,^23^ GaMo_4_Se_8_, AlMo_4_S_8_,^24^ GaV_4_S_8_,^25^ and GaV_4_Se_8_,^26^ a Jahn–Teller-type structural phase transition has been observed at low temperature (*T*_JT_) resulting in a distorted cell with a polar rhombohedral symmetry of space group *R*3*m*. In the Mo compounds, this distortion compresses the noncentrosymmetric cubic lattice along one of its four [111] axes, reducing the point symmetry of the B_4_ tetrahedra from *T*_*d*_ to *C*_3*v*_. The unit cell of the resulting phase in the rhombohedral setting is drawn in the upper branch of Figure 1. The rhombohedral cell is also drawn inside the cubic cell, where it can be seen that the cell volume is reduced to a quarter of its size and . The subscripts refer to the rhombohedral and cubic cells, respectively. At temperatures further below *T*_JT_, there is a second-order magnetic transition and these compounds present a rich magnetic phase diagram with several metamagnetic states and collinear ferromagnetic ordering when a critical external field is applied.^18,19,27^

To understand the origin of the physical properties observed in these compounds, in particular, the stabilization of a ferromagnetic ground state in the *R*3*m* phase, a molecular orbital approach is more appropriate rather than treating the ions as if they have localized spins. This is because the transition-metal ions are within such tight clusters and act like molecules within the solid, as described in a recent review.^28^ The electronic structure of a Mo_4_Se_4_ cubane unit is illustrated in the molecular orbital diagram in Figure 1. Direct d–d hopping occurs between the metal ions, which creates six bonding states from the 12 t_2g_ total orbitals per cluster. Additional hopping with p orbitals from the Se ions reduces the degeneracy of the bonding states to the *F*4̅3*m* scheme seen in Figure 1. Then, when the Jahn–Teller distortion occurs, the degeneracy of the t_2_ state reduces, creating an e doublet and an a_1_ bonding singlet, which is the highest occupied molecular orbital (HOMO). Whether the distortion is compressive (for Mo compounds) or elongative (for V compounds) changes which of the e and a_1_ states has higher energy, but the differing number of electrons in Mo and V clusters means that the a_1_ state is always occupied with one electron. In a Mo_4_ tetrahedron, there are 11 electrons in total.^29^ Thus, there is only *S* = 1/2 per tetrahedron, which is much lower than expected in a localized picture. Furthermore, the moment in one Mo_4_ unit interacts ferromagnetically with the moments in neighboring Mo_4_ units and is better described using an itinerant electron formalism.^27^ The noncentrosymmetric structure at low temperature results in competing antisymmetric exchange interactions and magnetocrystalline anisotropy, stabilizing noncollinear magnetic ground states and together with the strength of the spin–orbit coupling (SOC), control the exotic physical properties observed in these compounds. Thus, an in-depth understanding of the crystal structure is crucial to harness the functionalities offered by these compounds.

Recently, Schueller et al.^19^ performed synchrotron powder diffraction on polycrystalline GaMo_4_Se_8_ and observed that below *T*_JT_, the previously reported rhombohedral *R*3*m* phase coexists with a metastable orthorhombic phase indexed in the space group *Imm*2. This was justified with the possibility of two distortions with very similar formation energies taking place along two different order parameter directions (*a*, *a*, *a*) and (*a*, 0, 0). Both of these space groups are subgroups of *F*4̅3*m* and the lower branch of Figure 1 shows the orthorhombic unit cell. It is also drawn inside the cubic cell, where it can be seen that the cell volume is halved and , , and *c*_o_ = *c*_c_. The subscripts refer to the orthorhombic and cubic cells, respectively. Instead of the distortion occurring along the [111] axis (which happens for the *R*3*m* phase), the *Imm*2 phase results from distortions occurring along the [100] and [010] directions. This reduces the point symmetry of the B_4_ tetrahedra from *T*_*d*_ to *C*_2*v*_. Among lacunar spinels, only GeV_4_S_8_,^30^ GeV_4_Se_8_,^26^ and the Ge-rich members of Ga_1–*x*_Ge_*x*_V_4_S_8_^31^ have been found to distort to *Imm*2 instead of *R*3*m*. This can be understood as the HOMO degeneracy of the B_4_ cluster splits differently through the Jahn–Teller transition, due to the additional electron per B_4_ cluster.

Currently, there is an effort to understand the magnetic phase diagrams of the lacunar spinels known to have *R*3*m* symmetry below the structural transition. In GaMo_4_S_8_, a Néel-type skyrmion phase has been theoretically predicted^29^ but a modulated magnetic state with shorter periodicity (λ ≈ 9.8 nm) has been experimentally observed,^18^ compared to that of the observed skyrmion phases in GaV_4_S_8_ and GaV_4_Se_8_ (λ ≈ 20 nm). This is attributed to the larger SOC in the 4d Mo compounds compared to the 3d V compounds. In the compound we study here, GaMo_4_Se_8_, a skyrmion lattice has been predicted by computation,^32^ and Schueller et al.^19^ have identified a cycloidal phase and a Néel skyrmion phase (in a wide-phase field region), both with a periodicity of λ ≈ 16 nm, whereas the *Imm*2 phase is predicted to be a uniaxial ferromagnet, which highlights how a small distortion could have a drastic impact on the magnetic properties. Therefore, a detailed knowledge of the nuclear structure of each phases is required before understanding the subtleties of their respective magnetic structures.

The symmetry-mode approach is a powerful method to link physical properties to specific distortion modes (e.g., polarization in an improper ferroelectric).^33^ It is based on separating all of the atomic displacement and strain modes of the daughter low-symmetry structure allowed by the irreducible representations of the higher symmetry aristotype. In this study, we have used ISODISTORT^34^ to describe the low-temperature phases of GaMo_4_Se_8_ in terms of strain and atomic displacement modes. In Figure 1, the modes associated with both structures (rhombohedral and orthorhombic) are indicated and the overall effect of the distortions on the Mo_4_ tetrahedra is highlighted with arrows. The fact that only Γ modes (corresponding to the Brillouin zone center) are allowed means that all tetrahedra in each phase are affected in the same way. The Γ_1_ identity representation, preserving the symmetry of the parent phase for both low-temperature hettotypes, is essentially thermal expansion; all lattice parameters are affected equally. In the *R*3*m* phase of GaMo_4_Se_8_, the Γ_4_ strain mode is the one responsible for the tensile strain. It compresses the parent cubic cell along one of the [111] body diagonals resulting in rhombohedral angle values in excess of 60°. Its effect on the *R*3*m* cell in the hexagonal setting consists of the simultaneous cell compression along *c* and stretching along *a*. In the *Imm*2 phase of GaMo_4_Se_8_, the tensile strain is due to the Γ_3_ mode compressing the orthorhombic cell along *c* (aligned with one of the [100] axes of the parent cubic cell) and stretching it along *a* and *b*. The Γ_4_ strain mode results from applying shear strain to the opposite edges of the cubic cell driving its angles away from 90°. It changes the *a*/*b* length ratio of the orthorhombic *Imm*2 cell leaving the *c* axis intact. The effect of the Γ_3_ and Γ_4_ strain modes is visualized for both phases in the Supporting Information Videos. The effect of the atomic displacement modes on all atom positions in both phases is illustrated with arrows in Figure S1.

In this study, we have collected variable temperature powder neutron diffraction data on polycrystalline GaMo_4_Se_8_ and performed Rietveld refinement both for the high-temperature *F*4̅3*m* and low-temperature *R*3*m* and *Imm*2 phases. The refined structures were analyzed using ISODISTORT to calculate the magnitudes of the distortion modes across the structural transition. These are then compared to other known lacunar spinel compounds and a possible explanation of the origin of the phase coexistence is proposed. We have also grown single-domain crystals of GaMo_4_Se_8_, with which we have performed X-ray diffraction and measurements of its magnetism with variable temperature and applied magnetic field. From this, we carry out critical exponent analysis, which for lacunar spinels has so far only been reported for GaV_4_S_8_,^35^ finding that GaMo_4_Se_8_ cannot be described with a single universality class. We then also perform full magnetoentropic mapping analysis, in which evidence for the formation of a Néel skyrmion lattice phase is found.

## Experimental Section

### Polycrystalline Synthesis

In preparation for the synthesis of GaMo_4_Se_8_ powder, gallium pieces (Alfa Aesar, 7N) were added to a mixture of molybdenum (Alfa Aesar, 99.95%) and selenium (Sigma-Aldrich, ≥99.5%) powders. The synthesis protocol described by Schueller et al.^19^ was followed, in which the mixture (with 50% excess Ga over stoichiometry) is sealed in an evacuated quartz tube in a 1 g batch. It is then heated to 1283 K at a rate of 8 K/min, held for 20 h, and then quenched in cold water. Phase identification was performed using X-ray diffraction on a Rigaku SmartLab diffractometer with monochromatic Cu Kα radiation. This revealed large quantities of binary impurities indicating that the reaction was not complete. The powder was therefore annealed under the same conditions, which removed most of the impurity phases with only a small amount of Mo_3_Ga being observed. Three 1 g batches prepared in this way were combined and annealed twice more to produce a 3 g batch used to perform powder neutron diffraction.

### Single-Crystal Growth

For the growth of GaMo_4_Se_8_ single crystals, 2 g of the same elemental precursors as the polycrystalline synthesis was mixed and sealed in an evacuated quartz tube. An excess of Ga (a stoichiometry of Ga_15_Mo_26.3_Se_56.6_) was used. In the method by Yaich et al.,^36^ the tube is placed in a muffle furnace and heated at 1423 K for several days and then cooled at 2 K/h to 1073 K. In our attempt using these parameters, many red platelets of GaSe were also produced possibly because Mo has low mobility in the reaction mixture. Therefore, we used a dwell time of 14 days and cooled at 1 K/h to 1073 K, followed by a quench in cold water. This method increased the number of GaMo_4_Se_8_ crystals produced. Many intergrown clusters of black crystals of GaMo_4_Se_8_ grew within the charge as well as small single crystals with defined facets (approximately 0.5 × 0.5 × 0.5 mm, see Figure S2), which were mechanically extracted using tweezers.

### Single-Crystal Diffraction

Single-crystal X-ray diffraction data were collected on three different samples using two different diffractometers. All three samples were cut from single crystals and mounted on a loop. Crystal 1 was measured using a Bruker D8 VENTURE diffractometer with a Photon 100 detector at room temperature, and the data were collected using the Bruker APEX3 interface. Crystals 2 and 3 were cooled to 100 K in a Rigaku MicroMax-007 HF diffractometer with a Rigaku Saturn724+ detector, and CrystalClear 2.0 was used for data collection. Both diffractometers have Mo Kα (λ = 0.71073 Å) X-ray sources. Indexation, integration, and reduction of all data sets were carried out with CrysAlisPro.^37^ The empirical absorption correction was performed using spherical harmonics implemented in the SCALE3 ABSPACK scaling algorithm. Olex2^38^ with SHELXT^39^ was used for the structural solution, and Jana2006^40^ was used for refinement. Within Jana2006, averaging of the data was performed and the SHELX extinction method was used.

### Powder Neutron Diffraction

The powder neutron diffraction experiment^41^ was carried out on the high-resolution 2-axis diffractometer D2B at Institut Laue-Langevin, Grenoble. The neutron wavelength of λ = 1.594 Å was produced by reflection of the white beam of the [335] family of planes in a germanium monochromator. A total of 2.52 g of polycrystalline GaMo_4_Se_8_ was loaded into a cylindrical vanadium can of 4.7 mm in diameter. First, a diffraction pattern was collected at 300 K, well above the reported structural (51 K) and magnetic (27.5 K)^19,23^ transitions. Subsequently, the patterns were collected at temperatures of 65, 45, 40, 35, 30, and 2 K to study the evolution of the crystal structure at low temperature. The diffraction patterns were recorded in a diffraction angle range of 0.05–159.95° with a step of 0.05°. Rietveld refinements were carried out using FullProf^42^ program for all the diffraction patterns. The peak profile was modeled using a Thompson–Cox–Hastings pseudo-Voigt function convoluted with axial divergence to allow for the asymmetry. More details of the refinement process are provided in Supporting Information.

### Magnetic Measurements

The magnetic measurements were conducted in a Quantum Design MPMS 3 system. A small single crystal was fixed onto a quartz sample holder with low susceptibility varnish. DC measurements of the total magnetic moment *m* of the sample (in *Am*^2^) against external magnetic field *H* on increasing field from 0 to 7 T were collected at isotherms from 15 to 40 K in 1 K increments using the vibrating sample method. The critical exponents β and γ were determined by the Arrott–Noakes method^43^ using an iterative code. The magnetoentropic mapping was obtained by measuring the total magnetic moment *m* against *T* on heating from 15 to 40 K at a constant magnetic field *H* from 5 to 310 mT in 5 mT increments. Once collected, the data were input into a Python script made publicly available by Bocarsly et al.^44^

## Results and Discussion

### Single-Crystal Structure above the Structural Transition

The noncentrosymmetric symmetry (lack of an inversion center) in the cubic phase of GaMo_4_Se_8_ means that two inversion twins of the structure exist but their distinction is not trivial, as Friedel pairs (the couple of reflections *h*, *k*, *l* and *h̅*, *k̅*, *l̅*) appear identical due to the phase problem.^45^ To determine the absolute structure (the orientation of the noncentrosymmetric crystal), it is possible to take advantage of anomalous scattering to circumvent this problem.^46^ At the wavelength used in the single-crystal X-ray diffraction (λ = 0.71073 Å), there is a non-negligible imaginary component (*f*″) of the anomalous dispersion for Ga and Se (see Figure S3), breaking Friedel’s law and allowing us to distinguish the difference between the two inversion twins of the crystals used in the experiment, or if twinning is present, the proportion of each twin in the sample (expected to be a 50:50 mixture in total of inversion twins). The twinning matrix is the inversion symmetry operator, which has the effect of changing all atomic coordinates by 1 – *x* and the molar ratio of each twin can be quantified by the Flack parameter.^47^

Using this method, we were able to isolate three single crystals (labeled crystal 1–3 here) which are single domain. The atomic coordinates resulting from the crystal with the best fit (crystal 2, w*R* = 1.36) are displayed in Table 1 with a Flack parameter converging to a value of 0 (within error) after an initial guess of 1, showing that the absolute structure can be determined for this compound. In this table, it can be seen that in the cubic structure, there are four fully occupied Wyckoff positions, namely, Ga (4a), Mo1 (16e), Se1 (16e), and Se2 (16e). In our measurements, it was determined that crystals 2 and 3 present the same inversion twin as previously reported,^24^ whereas crystal 1 is the inversion twin of this structure. The atomic coordinates of crystals 1 and 3 are shown in Tables S1 and S2, where it can be seen that they are related to crystal 2 by 1 – *x* with a Flack parameter of 0, indicating that each of the crystals contains only one inversion domain. This result shows that the growth method presented in this paper can produce single inversion domain crystals.

**Table 1:** Crystal 2 Fractional Atomic Coordinates and Isotropic Equivalent Atomic Displacement Parameters in *F*4̅3*m* (100 K) with *a* = 10.1608(6) Å, *V* = 1049.02(11) Å^3^, and w*R*(obs) = 1.36, *R*(obs) = 0.86, GOF(obs) = 1.19, and Flack Parameter = 0.02(2)

label	Wyckoff	*x*	*y*	*z*	Occ.	*U*_iso_
Mo1	16e	0.39925(3)	0.39925(3)	0.39925(3)	1	0.00344(10)
Se1	16e	0.63624(5)	0.63624(5)	0.63624(5)	1	0.00467(14)
Se2	16e	0.13655(5)	0.13655(5)	0.13655(5)	1	0.00316(14)
Ga	4a	0	0	0	1	0.0049(3)

### Powder Neutron Diffraction through the Structural Transition

**Figure 2:**
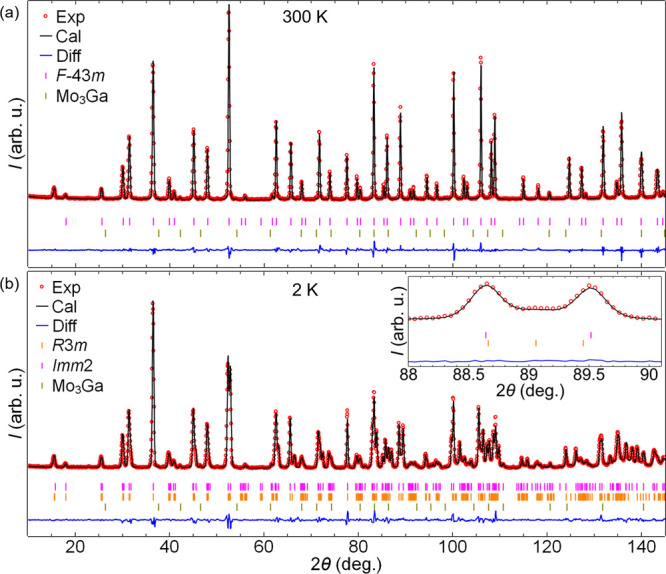
Observed (red open circles) and calculated (black solid line) powder neutron diffraction patterns measured at (a) 300 K, high-temperature phase (*F*4̅3*m*), and (b) 2 K, low-temperature phases (*R*3*m* and *Imm*2 Bragg reflections in magenta and orange vertical lines, respectively). The blue line is the difference plot. The inset in (b) highlights the region around the cubic (408) peak which splits through the Jahn–Teller transition with contributions from both *R*3*m* and *Imm*2 phases visible. A small presence of the impurity phase Mo_3_Ga is shown by olive vertical lines.

Figure 2 shows powder neutron diffraction patterns measured for the temperatures of 300 and 2 K. At 300 K (Figure 2a), the calculated profile using an *F*4̅3*m* phase fits excellently with the experimental pattern, which is consistent with the previous reports^19,23^ and our single-crystal refinement. The diffraction pattern of 65 K (Figure S4a) also fits very well with the same space group, indicating the same crystal structure as at 300 K. The atomic coordinates and cell parameters obtained from the 300 K diffraction data are presented in Table 2.

**Table 2:** Fractional Atomic Coordinates and Isotropic Equivalent Atomic Displacement Parameters for *F*4̅3*m* (300 K) with *a* = 10.17818(2) Å, *V* = 1054.412(4) Å^3^, and *R*_p_ = 4.195, w*R*_p_ = 5.59, and χ^2^ = 3.6

label	Wyckoff	*x*	*Y*	*Z*	Occ.	*U*_iso_
Mo1	16e	0.39927(7)	0.39927(7)	0.39927(7)	1	0.0033(2)
Se1	16e	0.63623(11)	0.63623(11)	0.63623(11)	1	0.0050(4)
Se2	16e	0.13645(11)	0.13645(11)	0.13645(11)	1	0.0032(4)
Ga	4a	0	0	0	1	0.0042(6)

When the temperature is lowered below 65 K, the pattern behavior changes and numerous split peaks [as illustrated by the 440 cubic reflection (Figure S5)] are observed. The peak splitting indicates that the high-temperature cubic structure undergoes a structural transformation below 65 K, which is in line with the previous studies^19,23^ reporting the phase transition to take place around 51 K. In Figure 2b, the diffraction pattern of 2 K is shown. We first performed Rietveld refinement on the 2 K diffraction pattern with an *R*3*m* crystal structure model. In this noncentrosymmetric space group, the Ga is now on the general position 1*a* which is set at (0,0,0) to define the origin and the polar axis is along the 111 direction as it contains the threefold rotation axis. However, this refinement leads to several unindexed peaks in the pattern (Figure S6), indicating that there might be another coexisting phase in the sample. We then considered a model including both *R*3*m* and *Imm*2 phases as proposed by the powder synchrotron study^19^ and as it can be seen in Figure 2b, the model fits the experimental pattern excellently. The *Imm*2 space group is also noncentrosymmetric and Ga is on a 2a (0,0,*z*) position. The polar axis in this orthorhombic phase has to be along the twofold rotation axis, thus along *z* and we fixed *z* = 0.5 as derived from the high-symmetry structure. The peak positions for both phases are very close to each other, but the additional peaks corresponding to *Imm*2 can be discerned at several angles (see inset of Figure 2b). For all other diffraction patterns (30, 35, 40, and 45 K) below the structural transition (*T*_JT_), the combined model of *R*3*m* and *Imm*2 phases fits excellently (Figure S4b–e). A tiny fraction (≈1%) of an impurity phase, identified as Mo_3_Ga, is also detected in the sample as seen in Figure 2. In Tables 3 and 4, the Wyckoff and atomic positions in the unit cell along with isotropic displacement parameters are presented for the *R*3*m* and *Imm*2 phases, respectively.

**Table 3:** Fractional Atomic Coordinates and Isotropic Equivalent Atomic Displacement Parameters for *R*3*m* (2 K) with *a* = 7.15251(4) Å, α = 60.6404(7)°, *V* = 262.481(8) Å^3^, and *R*_p_ = 4.04, w*R*_p_ = 5.495, and χ^2^ = 4.36

label	Wyckoff	*x*	*Y*	*z*	Occ.	*U*_iso_
Mo1	1a	0.4019(3)	0.4019(3)	0.4019(3)	1	0.0053(12)
Mo2	3b	0.3971(4)	0.8064(4)	0.3971(4)	1	0.0014(5)
Se1	1a	0.6355(4)	0.6355(4)	0.6355(4)	1	0.0041(11)
Se2	3b	0.6394(4)	0.0905(5)	0.6394(4)	1	0.0026(8)
Se3	1a	0.1367(3)	0.1367(3)	0.1367(3)	1	0.0001(1)
Se4	3b	0.1381(4)	0.5911(5)	0.1381(4)	1	0.0032(8)
Ga	1a	0	0	0	1	0.0003(8)

**Table 4:** Fractional Atomic Coordinates and Isotropic Equivalent Atomic Displacement Parameters for *Imm*2 (2 K) with *a* = 7.21956(19) Å, *b* = 7.1560(5) Å, *c* = 10.1649(3), *V* = 525.21(4) Å^3^, and *R*_p_ = 4.04, *wR*_p_ = 5.495, and χ^2^ = 4.36

label	Wyckoff	*X*	*Y*	*Z*	Occ.	*U*_iso_
Mo1	4d	0	0.807(2)	0.896(3)	1	0.005(3)
Mo2	4c	0.791(3)	0	0.101(3)	1	0.012(4)
Se1	4d	0	0.264(2)	0.141(3)	1	0.001(3)
Se2	4c	0.286(3)	0	0.869(3)	1	0.020(4)
Se3	4d	0	0.281(2)	0.641(3)	1	0.005(3)
Se4	4c	0.274(3)	0	0.370(3)	1	0.015(5)
Ga	2a	0	0	0.5	1	0.020(4)

**Figure 3:**
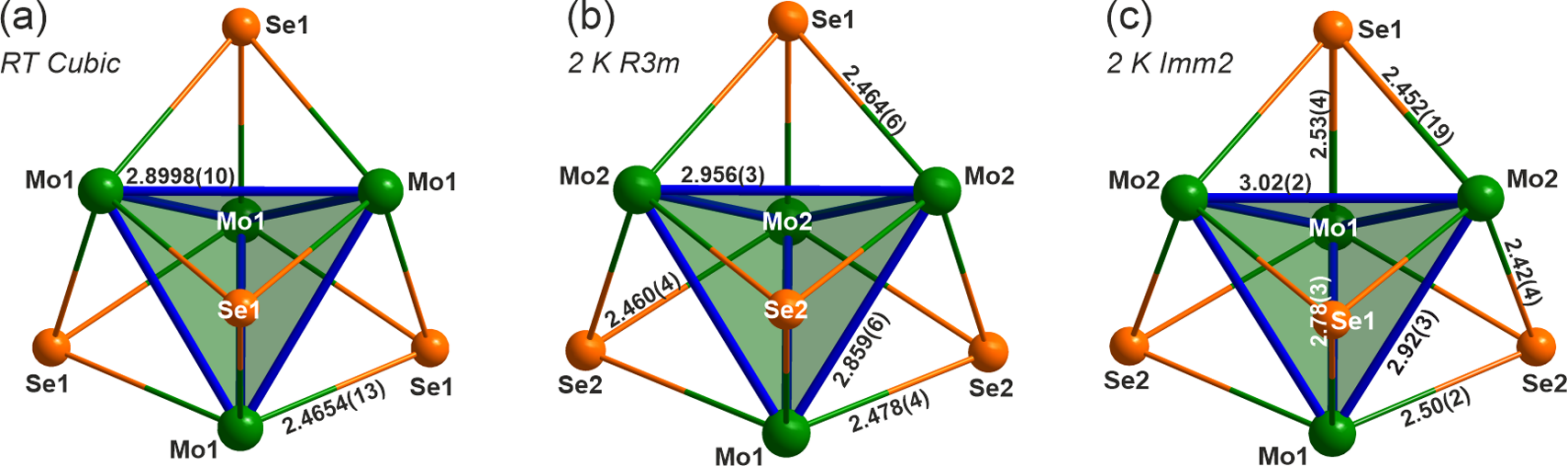
Mo_4_Se_4_ clusters as obtained from the powder neutron diffraction refinements, of the (a) *F*4̅3*m* phase at room temperature, (b) *R*3*m* phase at 2 K, and (c) *Imm*2 phase at 2 K. Bonds are labeled with distances (in Å) and atoms are labeled with the labels used in Tables 2–4, respectively. The Mo–Mo bonds are highlighted in blue.

The Rietveld refinement outlined above allows us to investigate changes in the crystal chemistry through the phase transition. In Figure 3, the Mo_4_Se_4_ cubane clusters are drawn for the cubic, rhombohedral, and orthorhombic phases, with bond lengths being included. The cubic and rhombohedral bond lengths here are similar to what has been previously reported in GaMo_4_Se_8_^24^ and the same thing happens through the structural transition; the Mo2–Mo2 distances increase. The same happens in GaMo_4_S_8_,^23^ but in that compound, the equivalent bond lengths are shorter and so the Mo cations in its cubane units are slightly more tightly packed. In the *Imm*2 phase, the change in symmetry alters the number of nonequivalent bond lengths. The contrast between Mo1–Mo1 and Mo2–Mo2 distances and therefore how much the atoms move are visible in Figure 3c, with lengths of 2.78(3) and 3.02(2) Å, respectively. It is also worth noting that the Mo–Mo distances in this compound are much shorter, for example, than within the layers in MoSe_2_ (3.29 Å).

**Figure 4:**
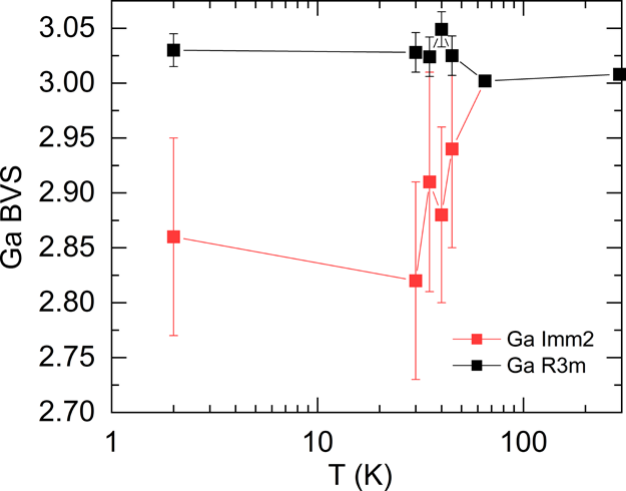
Calculated BVS of the Ga ions in GaMo_4_Se_8_ as a function of temperature. The error bars for *Imm*2 are much larger due to the larger errors in the atomic positions for that phase.

The bond valence sum (BVS) of each ion can also be calculated, which can infer information about the possible charge transfer in the system. The BVS rule states that the sum of bond valences around an ion is equal to its atomic valence.^48^ The bond valence, *S*_*ij*_, of each bond is approximated by
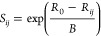
1where *R*_*ij*_ is the distance between the two atoms *i* and *j* and *R*_0_ and *B* are parameters depending on the ions involved. All Ga–Se and Mo–Se bonds with a cutoff length of 2.6 Å and the values of *R*_0_ and *B* recommended by Brese and O’Keeffe^49^ were accounted for in the calculations. The BVS of the Ga ions (Figure 4) and Mo ions (Figure S8) has been calculated as a function of temperature. The expected valencies (Ga^3+^ and Mo^3.25+^) are obtained above the structural transition. There is a noticeable drop in the BVS of Ga in the *Imm*2 phase below the transition, whereas for the *R*3*m* phase, it remains close to the expected value of 3. This suggests that the bonding in the GaSe_4_ tetrahedra is altered differently in each of the phases and that the transition to *Imm*2 may be accompanied with an electronic instability such as the long-range electronic phase separation observed in mixed valence manganite perovskites^50^ and CaFe_3_O_5_,^51^ where phase coexistence together with a change in the BVS through the structural transition was observed.

**Figure 5:**
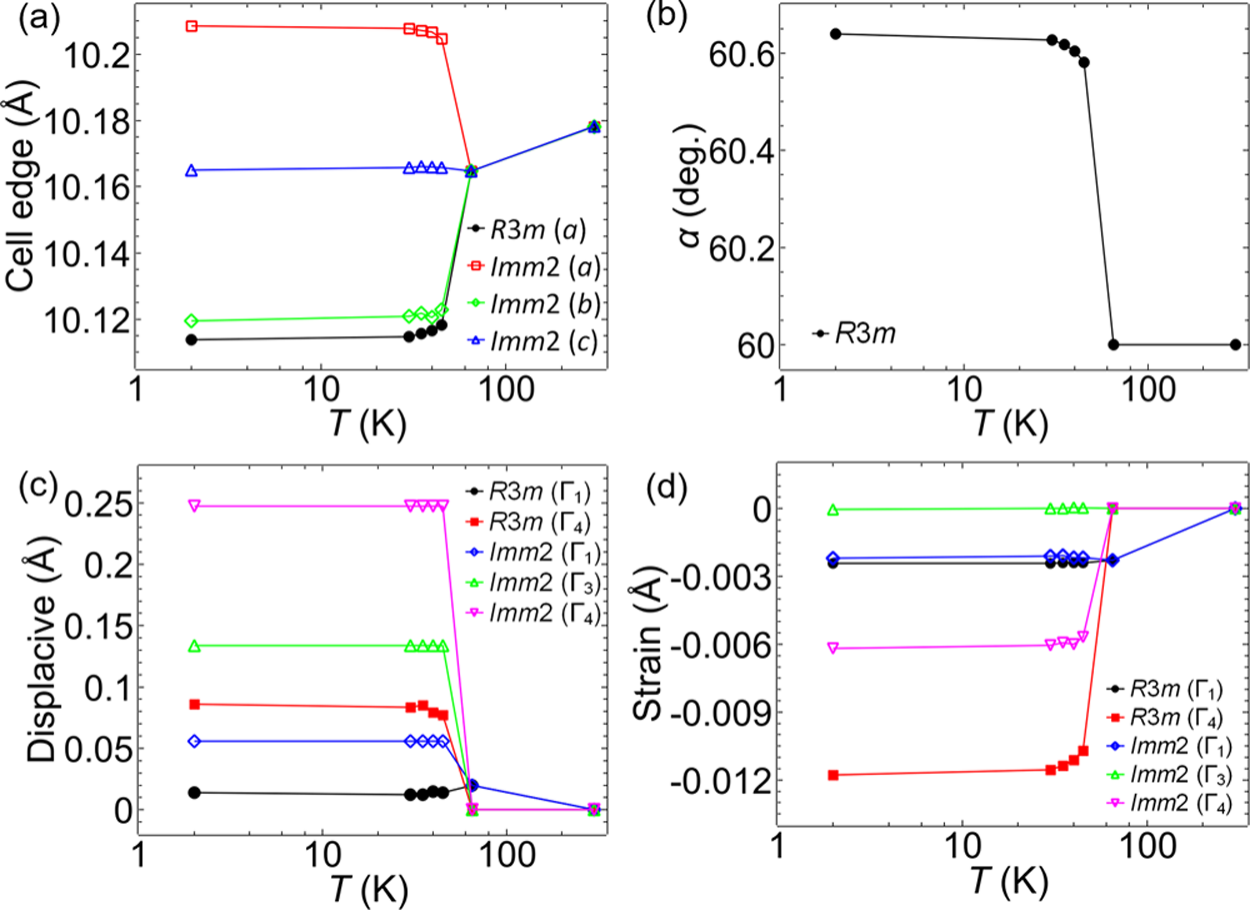
(a) Cell edge, (b) rhombohedral angle, (c) displacive, and (d) strain modes as a function of temperature. The room-temperature crystal structure is used in comparison with the daughter phases to calculate the distortion modes.

The cell edge variations for the cubic (*F*4̅3*m*), rhombohedral (*R*3*m*), and orthorhombic (*Imm*2) phases are presented in Figure 5a. The pseudo-cubic lattice parameters (defined in Figure 1) are used to compare the two phases. As can be seen in Figure 5a, for the *R*3*m* phase, a sharp decrease is observed between 45 and 65 K, due to the structural transition at 51 K. The large change observed in *a*_r_ is accompanied with a change in rhombohedral angle (α), as shown in Figure 5b. α varies from 60° at 300 K to 60.6404(7)° at 2 K corresponding to a 1.067% change. It is worth noting that the percentage change in the rhombohedral a_r_ and α is the largest for GaMo_4_Se_8_ among the lacunar spinel compounds (Figure S9a,b), indicating the largest extent of distortion. The evolution of cell volume (Figure S10a) and phase percentage (Figure S10b) is described in Supporting Information. In the *Imm*2 phase, a small increase in the cell volume is observed just below the phase transition which is primarily due to an elongation along *a*_o_ supporting the hypothesis that this second phase appears to counteract the large compression of the rhombohedral phase.

In order to rationalize these observations, we have decomposed the distortion between phases into their normal modes.^33^ We present the atomic displacement (Figure 5c) and strain component (Figure 5d) of the distortion modes Γ_1_ and Γ_3_ (only present in the *Imm*2 phase) and Γ_4_ (as illustrated in Figure 1) calculated for the two phases as a function of temperature. In order to calculate the amplitude of the modes, the room-temperature (*F*4̅3*m*) structure was considered as the parent cell and all the low-temperature structures (*F*4̅3*m*, *R*3*m*, and *Imm*2) were considered daughter cells. In Figure 5c, the overall normalized magnitude of the displacive distortion modes (on all atoms) is shown.^34^ Above the phase transition (*T*_JT_), only Γ_1_ is nonzero. For temperatures below *T*_JT_, Γ_1_ changes oppositely for the rhombohedral (*R*3*m*) and the orthorhombic (*Imm*2) phases, as is also observed in the variation of the lattice parameters. The sharp change in cell volumes and distortion mode magnitudes in a narrow temperature range suggests that the transition is first-order and displacive in nature which is consistent with the very large peak observed in the specific heat.^27^ The magnitude of Γ_4_ for both phases is the largest and therefore, considering Landau phase transition theory, is likely to be the order parameter.^33^ For the *Imm*2 phase, both Γ_3_ and Γ_4_ displacive magnitudes are larger than in the *R*3*m* phase. The amplitudes of the atomic displacement modes of each atom are listed in Tables S5 and S6 and are shown graphically with directional arrows in Figure S1.

In Figure 5d, the strain is shown and similarly to the displacive amplitudes, at 65 K, only the Γ_1_ mode is nonzero. Below *T*_JT_, the variation in Γ_1_ (*R*3*m*) and Γ_1_ (*Imm*2) is similar and much smaller than Γ_4_. The Γ_3_ (*Imm*2) strain is almost zero throughout the temperature range, in contrast to the Γ_3_ displacement which is significant. The Γ_4_ strain for *R*3*m* and *Imm*2 decreases rapidly below *T*_JT_, followed by a slower decrease down to 2 K. The magnitude of the Γ_4_ strain of the *R*3*m* phase is almost twofold larger than that of the *Imm*2 phase. Energy landscape calculations have shown that the *R*3*m* phase is the ground-state structure, but the large strain amplitude observed in the *R*3*m* phase could stabilize the metastable *Imm*2 structure as observed in other spinel compounds^52^ and is consistent with the fact that GaMo_4_Se_8_ presents the largest strain mode among the lacunar spinel family (Figure S11b). This effect might be incipient in other systems such as a GaMo_4_S_8_ where the diffraction pattern shows strong anisotropic broadening at low temperature.^53^ In our diffraction pattern, the anisotropic microstrain tensor of the *R*3*m* phase from the 2 K refinement (Figure S12a) is largely directional along the c axis of the hexagonal cell (one of the [111] axes in the pseudo-cubic cell), whereas the *Imm*2 microstrain (Figure S12b) is more isotropic. This further supports the idea that internal strain in the *R*3*m* phase is correlated with the formation of the *Imm*2 phase, which is reminiscent to the accommodation strain mechanism in a martensitic transition, and together with the change in BVS in the *Imm*2 phase, suggests that this compound could host phenomena similar to the strain-induced metal–insulator phase coexistence observed in perovskite manganites,^54,55^ such as colossal magnetoresistance.

### Critical Behavior Analysis

**Figure 6:**
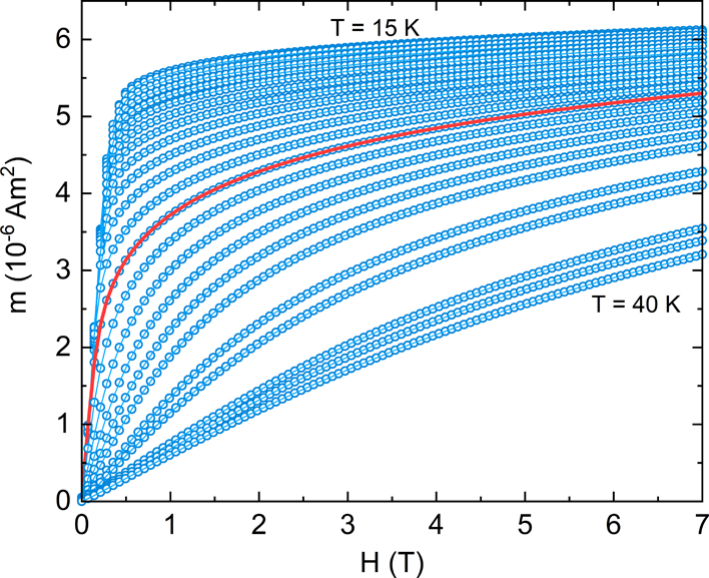
Measured total moment *m* (*Am*^2^) vs *H* (T) at isotherms from 15 to 40 K. The closest isotherm to the *T*_c_ is 27 K, indicated with the red curve.

Critical behavior analysis, where the magnetic ordering exponent β, the susceptibility exponent γ, and the critical isotherm exponent δ are determined, can be used to gain a better understanding of the magnetic interactions and nature of the ordering in magnets and has been used to identify the tricritical point in the magnetic phase diagram of the Néel-type skyrmion host GaV_4_S_8_.^35^ The isothermal magnetization data collected on a GaMo_4_Se_8_ single crystal around the *T*_c_ are presented in Figure 6, showing the transition from a ferromagnetic to a paramagnetic state and presents a similar behavior to previous reports with signs of metamagnetic states at a low magnetic field.^27^

**Figure 7:**
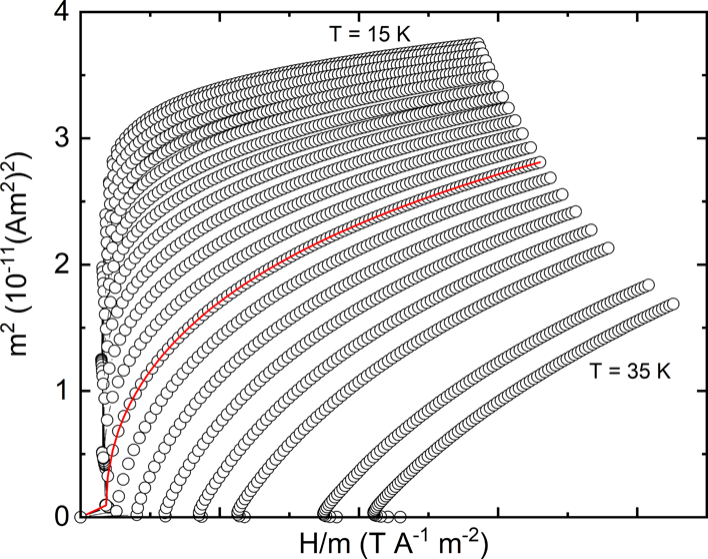
Arrott plot (mean-field model, β = 0.5 and γ = 1) with isotherms from 15 to 35 K being visible. The red curve indicates the 27 K isotherm.

Using these data, a conventional Arrott plot^56^ is presented in Figure 7, in which, according to the Banerjee criterion,^57^ the positive gradient of the isotherms at high field is indicative of a second-order magnetic transition. However, the slopes of the high field section in this plot are not parallel to each other suggesting that the magnetic transition in GaMo_4_Se_8_ is not described by the mean-field theory (where β = 0.5 and γ = 1.0). To test other universality classes, modified Arrott–Noakes plots^43^ have been produced using the equation of state
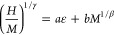
2where *a* and *b* are constants and ε = (T – *T*_c_)/*T*_c_ is the reduced temperature.

**Figure 8:**
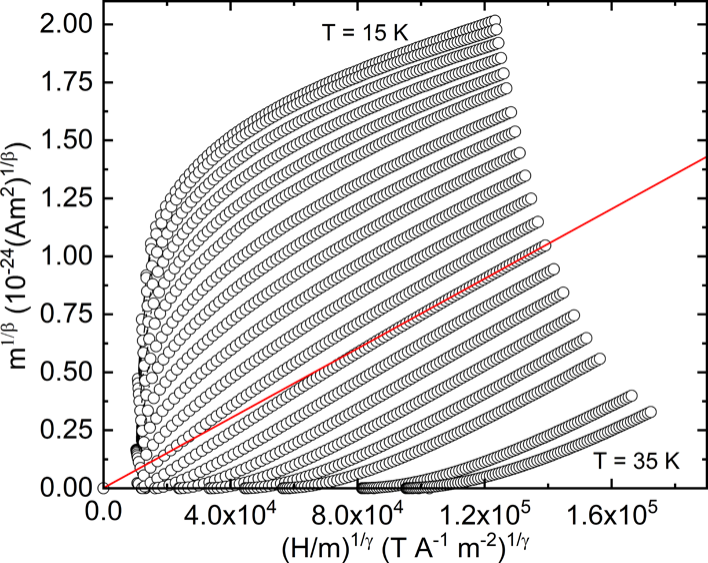
Arrott–Noakes plot using the best obtained critical exponents: β = 0.22 and γ = 1.19. The linear red line has been fit to the high field data points (*H* = 5.6–7 T) in the 27 K isotherm and extrapolated.

**Figure 9:**
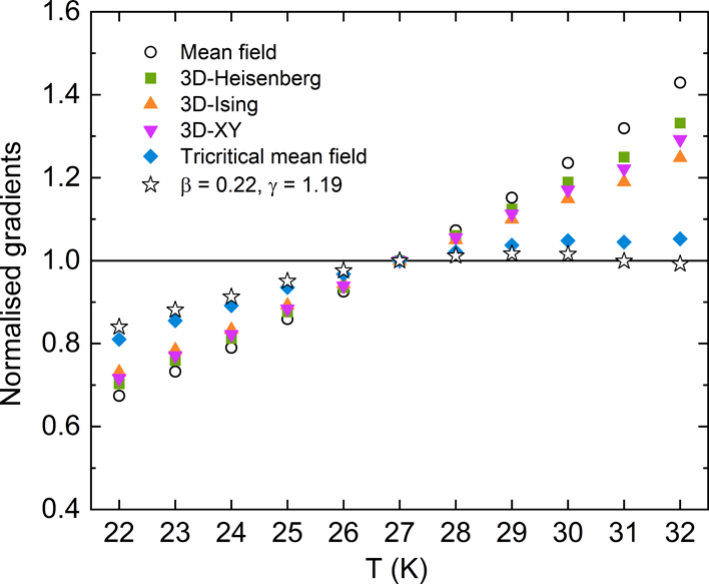
Gradients of linear fits to the high field data (*H* = 5.6–7 T) of each isotherm from modified Arrott plots from various theoretical models. The gradients have been normalized against the 27 K isotherm for each model.

Arrott–Noakes plots with exponents from some established theoretical models (3D-Ising, 3D Heisenberg, 3D-XY, and tricritical mean-field) are plotted in Figure S13. Of these models, the tricritical mean-field presents the most parallel lines immediately around *T*_c_. However, the line for the *T* = *T*_c_ = 27 *K* isotherm does not pass through the origin as expected from the equation of state, suggesting that the tricritical model does not describe the transition fully. Therefore, an iterative code was used to find a pair of critical exponents (β, γ) that fit both criteria and the refined values obtained using this method are β = 0.22(4) and γ = 1.19(1). The resulting Arrott–Noakes plot is shown in Figure 8, where the *T* = *T*_c_ = 27 K isotherm intercept passes close to the origin and the gradients at high field are overall more parallel than in the tricritical mean-field model, as shown in Figure 9.

**Figure 10:**
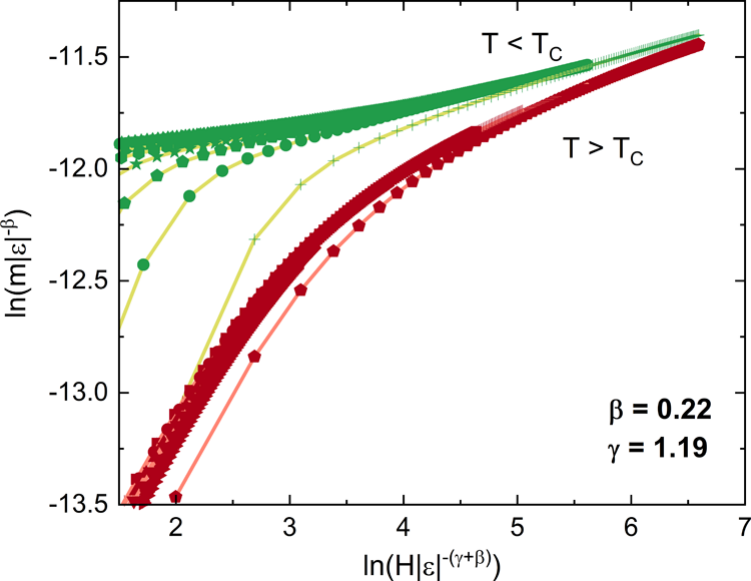
Scaling plot of the natural logarithms of the renormalized moment vs the renormalized field when β = 0.22 and γ = 1.19. The isotherms above 27 K are in red and those below 27 K are in green.

An alternative approach to test the validity of the critical exponents obtained using the Arrott–Noakes method is to express the magnetic equation of state in the critical region as
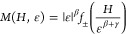
3where *f*_+_ and *f*_–_ are regular functions that apply for *T* > *T*_c_ and *T* < *T*_c_, respectively. Equation 3 implies that the renormalized moment (*m*|ε|^–β^) as a function of the renormalized field (*H*|ε|^–(β+γ)^) produces two universal curves for the isotherms above and below *T*_c_. Using the critical exponents obtained from the Arrott–Noakes plot above, the scaling plot is presented in Figure 10 showing that the isotherms are separated into two distinct groups. The deviation observed at low field in Figure 10 for all *T* < *T*_c_ isotherms is similar to what is observed in the GaV_4_S_8_ critical exponent report,^35^ where the change in gradient of the linear isotherm lines marks the transition between skyrmionic and ferromagnetic states. At the isotherm closest to the *T*_c_, this then marks the tricritical point between the skyrmionic, ferromagnetic, and paramagnetic states and this observation suggests that similar phases would be observed in GaMo_4_Se_8_.

The validity of the critical exponent can be further confirmed using the Widom scaling relation which states that γ = β(δ – 1), where the critical exponent δ can be experimentally obtained from the isothermal magnetization at *T* = *T*_c_ using the relation *m* ∝ *H*^1/δ^. Using this relation, we obtain an experimental value of δ = 6.42(1) (Figure S14) which is close to the expected value (6.41) calculated using the Widom relation with the β and γ being used in Figure 8.

The critical exponents obtained for GaMo_4_Se_8_ are close to what is expected for a tricritical mean-field magnet, in particular, the ordering exponent β (below *T*_c_) is equal to that of GaV_4_S_8_ which has been found to belong to the tricritical mean-field universality class.^35^ However, the value obtained for the susceptibility exponent γ (above *T*_c_) is higher than in GaV_4_S_8_ suggesting that GaMo_4_Se_8_ cannot be categorized with a single universality class. MnSi, a Bloch-type skyrmion compound, exhibits tricritical mean-field behavior with two different values for γ (0.915(3) on a single-crystal sample^58^ and 1.20(1) on a polycrystalline sample^59^) which is similar to the difference seen here between GaMo_4_Se_8_ and GaV_4_S_8_. The difference in MnSi was attributed possibly to magnetic scattering from crystal boundaries,^58^ which may also be the case here, as not only does GaMo_4_Se_8_ possess two distinct magnetic structures in the temperature range studied but we also observed that the crystal fractured into several pieces during our measurements, which could be due to the large strain created in the sample through the structural phase transition. This can be further probed by examining the exchange interaction distance *J*(*r*). The renormalization group approach suggests that long-range attractive interactions between spins decays as *J*(*r*) ≈ *r*^–(*d*+σ)^ where *r* is the distance between spins, *d* = 3 is the number of spatial dimensions, and σ is a positive constant that is related to γ.^60^ For GaMo_4_Se_8_, σ ≈ 1.75 is calculated and therefore *J*(*r*) ≈ *r*^–4.75^. This is markedly different from GaV_4_S_8_, for which σ = 1.316(4) and *J*(*r*) ≈ *r*^–4.3^.^35^ As *J*(*r*) is longer ranged in GaMo_4_Se_8_, it can be said to be in an intermediate range,^61^ which is more toward a 3D Heisenberg behavior (in which *J*(*r*) decreases faster than *r*^–5^) rather than purely mean-field (in which *J*(*r*) decreases slower than *r*^–4.5^) like GaV_4_S_8_ is but does not completely obey any of the established universality classes. Considering that two structures are coexisting and that both have been predicted to have different magnetic ground states (uniaxial ferromagnet for the *Imm*2 phase and cycloid for the *R*3*m* phase^19^), it is not surprising that the two magnetic structures present in GaMo_4_Se_8_ belong to different universality classes.

### Magnetoentropic Mapping

**Figure 11:**
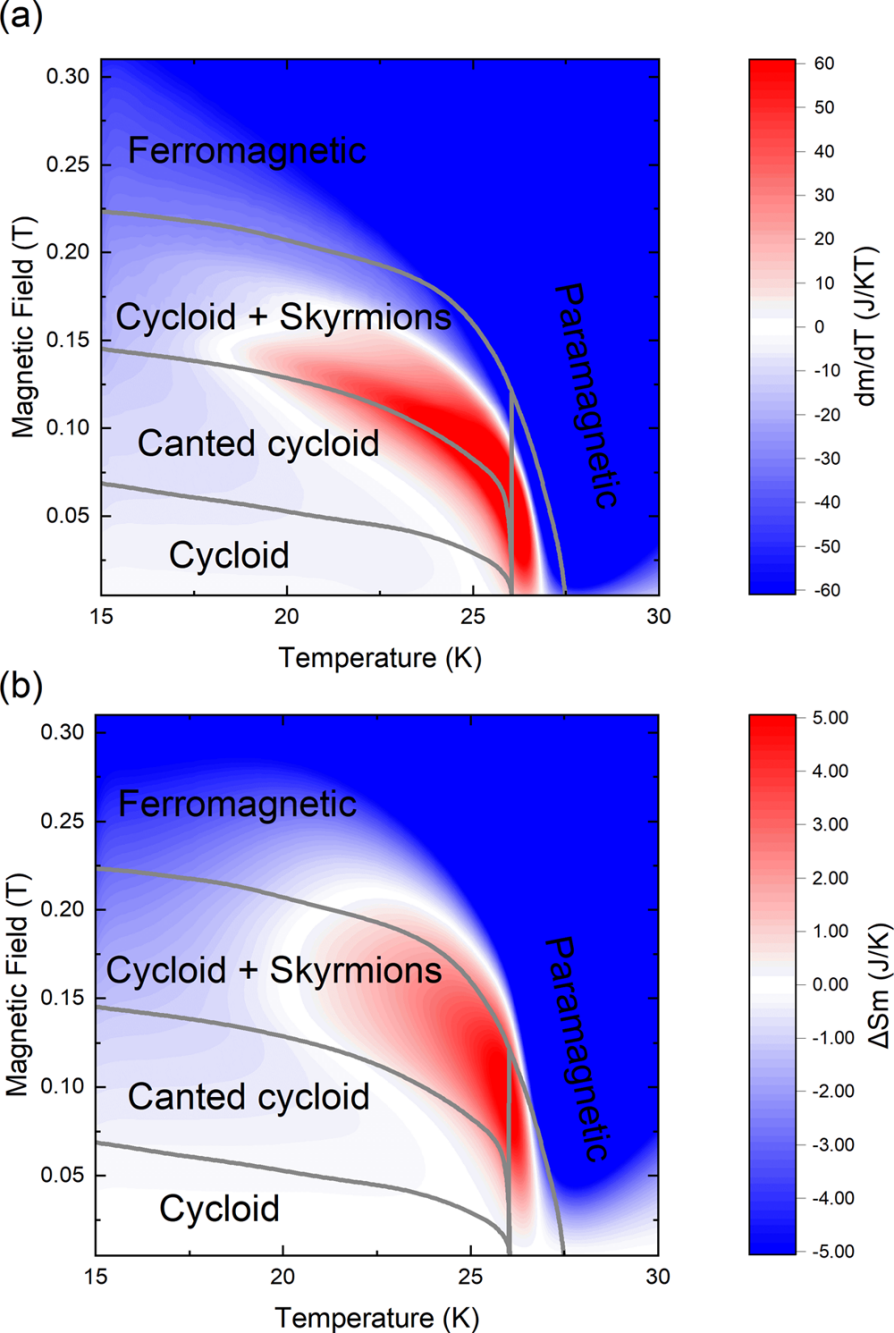
(a) Graphical mapping of (∂*m*/∂*T*)_H_ for various magnetic fields. (b) Map of Δ*S*_M_(*T*,*H*). The gray lines are reproduced from the magnetic phase diagram obtained from a polycrystalline sample.^19^

The magnetic phase diagram of polycrystalline GaMo_4_Se_8_ has previously been investigated showing several transitions from cycloidal states to a skyrmion phase to collinear ferromagnetism.^19^ Using temperature-dependent magnetization measurements at various applied fields (Figure S15) on a single crystal, we generated susceptibility (∂*m*/∂*T*)_H_ (Figure 11a) and magnetoentropic (∂*S*/∂*H*)_T_ (Figure 11b) maps. This procedure has been shown to highlight field-driven magnetic phase transitions, with skyrmion phases presenting a positive entropy phase field.^44^

In the map presented in Figure 11a, the high field region presents a negative first derivative (blue phase field), corresponding to the conventional magnetocaloric effect expected for a ferromagnet. At lower field, a phase field presenting a positive first derivative (red) indicates a field-induced phase transition. Using this representation and extracting the minima and maxima in the susceptibility, a phase diagram similar to the one obtained previously can be obtained.^27^ By integrating the susceptibility over the magnetic field, the features around the field-induced transition are highlighted by the change in entropy Δ*S*_M_, as presented in Figure 11b. This results in white phase field with Δ*S*_M_ ≈ 0, corresponding to magnetic states (cycloids) that can be stabilized without a change in entropy from zero field. The red Δ*S*_M_ phase field observed below the magnetic ordering temperature corresponds to an increase in entropy and is where the skyrmion phase is expected when the magnetic field is applied along the *c* axis. Here, the positive entropy phase field spans 5 K and 100 mT within the cycloidal phase, which is significantly different from the predicted skyrmion stability down to low temperature.^19^ This reduced temperature stability range is predicted for the GaMo_4_S_8_ phases when the intensity of Hund coupling is increased as the single ion energy increases.^29^

## Conclusions

In summary, we have performed detailed structural characterization with high-resolution powder neutron diffraction and single-crystal X-ray diffraction on high-quality samples of GaMo_4_Se_8_. The Rietveld refinement confirms the presence of two coexisting phases with rhombohedral and orthorhombic crystal structures at low temperature (<51 K), which is unique to GaMo_4_Se_8_ among the lacunar spinel family. We showed that the strain modes play an important role in the possible stabilization of the metastable orthorhombic phase and the recent observation of a metal–insulator transition in the pressure-induced orthorhombic phase of GaV_4_S_8_^62^ motivates further electrical characterization of the mixed phase observed in this Mo compound. In addition to that, magnetic measurements were carried out on a single-crystal sample. The critical exponent analysis shows that the compound primarily exhibits tricritical behavior as observed for other skyrmion hosting compounds and the magnetic phase diagram is complex, possibly due to the presence of various magnetic interactions. A phase field presenting a positive entropy change is found to exist in the (*T*, *H*)-phase diagram where the expected skyrmion lattice is expected to be. Therefore, our current study provides an excellent framework for the better understanding of lacunar spinel compounds as well as skyrmion hosting systems. The details obtained from the magnetoentropic study on our single crystals will be vital to perform further experiments to isolate the skyrmion phase and unearth the interesting magnetic structure that may be useful in the field of spintronics/skyrmionics. The possibility to observe the two phases using high-resolution powder diffraction offers the opportunity to understand the magnetism of the orthorhombic phase which is predicted to be a simple uniaxial ferromagnet and would allow disentangling the role of a foreign magnetic phase in the stabilization of a skyrmion lattice.

## Supporting Information Available

Schematic of role of distortion modes on atoms, optical images of single crystals of GaMo_4_Se_8_, powder neutron diffraction patterns at different temperatures, 2 K diffraction pattern fitting only with the *R*3*m* phase, displacement ellipsoids, BVS of Mo ions, percentage change in the cell parameters of lacunar spinel compounds, cell volume and phase percentage as a function of the temperature of GaMo_4_Se_8_, distortion modes of lacunar spinel compounds, 3D plots of anisotropic strain tensors, Arrott–Noakes plots, logarithmic magnetization plot at the *T*_c_, and temperature-dependent magnetization at various external fields (PDF)
Crystallographic information of single-crystal diffraction GaMo_4_Se_8__CRYSTAL1, deposition number 2071067 (CIF)
Crystallographic information of GaMo_4_Se_8__CRYSTAL2, deposition number 2071068 (CIF)
Crystallographic information of GaMo_4_Se_8__CRYSTAL3, deposition number 2071069 (CIF)
Crystallographic information of powder neutron diffraction 2K_Rhom_Orth-GaMo_4_Se_8__Mo_3_Ga, deposition number 2071070 (Rhom), 2071071 (Mo3Ga), and 2071072 (Orth) (CIF)
Crystallographic information of 300K_Cub-GaMo_4_Se_8__Mo_3_Ga, deposition number 2071073 (Cub) and 2071074 (Mo3Ga) (CIF)
Crystallographic information of 30K_Rhom_Orth-GaMo_4_Se_8__Mo_3_Ga, deposition number 2071075 (Rhom), 2071076 (Mo3Ga), and 2071077 (Orth) (CIF)
Crystallographic information of 35K_Rhom_Orth-GaMo_4_Se_8__Mo_3_Ga, deposition number 2071078 (Rhom), 2071079 (Mo3Ga), and 2071080 (Orth) (CIF)
Crystallographic information of 40K_Rhom_Orth-GaMo_4_Se_8__Mo_3_Ga, deposition number 2071081 (Rhom), 2071082 (Mo3Ga), and 2071083 (Orth) (CIF)
Crystallographic information of 45K_Rhom_Orth-GaMo_4_Se_8__Mo_3_Ga, deposition number 2071084 (Rhom), 2071085 (Mo3Ga), and 2071086 (Orth) (CIF)
Crystallographic information of 65K_Cub-GaMo_4_Se_8__Mo_3_Ga, deposition number 2071087 (Cub) and 2071088 (Mo3Ga) (CIF)
GaMo_4_Se_8__R3m_Gamma4 (MP4)
GaMo_4_Se_8__Imm2_Gamma3 (MP4)
GaMo_4_Se_8__R3m_Gamma4 (MP4)

## Supplementary Material

cm1c01448_si_001.pdf

cm1c01448_si_002.cif

cm1c01448_si_003.cif

cm1c01448_si_004.cif

cm1c01448_si_005.cif

cm1c01448_si_006.cif

cm1c01448_si_007.cif

cm1c01448_si_008.cif

cm1c01448_si_009.cif

cm1c01448_si_010.cif

cm1c01448_si_011.cif
