# Supplementary material for: Mode Crystallography Analysis through the Structural Phase Transition and Magnetic Critical Behavior of the Lacunar Spinel GaMo4Se8
Source: Chem Mater. 2021 Jul 6;33(14):5718–29. doi: 10.1021/acs.chemmater.1c01448 (PMC8382239; doi:10.1021/acs.chemmater.1c01448)
Supplement: Supplementary file 1 — cm1c01448_si_001.pdf [file cm1c01448_si_001.pdf]

Supporting Information

Mode crystallography analysis through the structural phase transition and magnetic critical behavior of the lacunar spinel $GaMo_4Se_8$

*Kieran Routledge, Praveen Vir, Nicholas Cook, Philip A. E. Murgatroyd, Sheikh J. Ahmed, Stanislav N. Savvin, John B. Claridge and Jonathan Alaria**

1 Supporting Text

1.1 Powder neutron diffraction refinement details

In the 300 K refinement, the sample contained a small amount (1%) of the impurity phase of Mo_3Ga. We were able to refine the thermal parameters anisotropically. The resultant anisotropic thermal parameters along with standard deviations are presented in Table S3. Considering these anisotropic thermal parameters, displacement ellipsoids are drawn and shown in Figure S7 for (a) the heterocubane Mo_4Se_4 unit and (b) the $GaSe_4$ tetrahedron. As it can be seen, the Se atoms have a large deviation from the ideal sphere, indicating a relatively large extent of thermal vibration of Se atoms in the crystal structure. For the Mo atoms, the displacement ellipsoid shape is close to the sphere, mainly due to the larger atomic weight of the element. No anisotropic thermal vibration is observed for the Ga atoms as it is at the high-symmetry position $4a$ (0,0,0). However, for all the low temperature patterns (below T_{JT}), where three phases are already included ($R3m$, $Imm2$, and Mo_3Ga), it was not feasible to refine the thermal parameters anisotropically.

As described in the main text, these patterns provide a good fit with the combined model of the $R3m$ and $Imm2$ phases. However, a close examination of peak shapes indicates that the strain might have a great influence on the phase separation in the sample. Therefore, we used Stephens' anisotropic peak broadening[1] for both the $R3m$ and $Imm2$ phases (with Laue class of strain tensors as -3m1 and mmm respectively) to improve the pattern fitting. The resulting strain tensors are listed in Table S4 and 3D plots of these anisotropic strain tensors are displayed in Figure S12.

1.2 Cell volume and phase percentage evolution

In Figure S10(a), the volume (converted to pseudo-cubic cell volume as illustrated in Figure 1 in the main text) is plotted as a function of temperature. The unit cell volume for the cubic ($F\bar{4}3m$) phase decreases monotonically from 300 to 65 K. Whereas, for the low-temperature structures (below T_{JT}), the volume is found to be slightly greater for the $Imm2$ phase. Figure S10(b) shows the change in the percentage of each phase present with temperature. As pointed out before, the compound crystallizes in the cubic structure above 51 K. Therefore, the phase percentage above 65 K, is 100, 0, 0 for the $F\bar{4}3m$, $R3m$, and $Imm2$ phases respectively. Below the structural transition, the cubic phase ($F\bar{4}3m$) transforms to rhombohedral ($R3m$) and orthorhombic ($Imm2$) with phase percentages of approximately 70% and 30%, respectively, which is consistent with the synchrotron data[2]. The variation in the phase percentage is constant below T_{JT}.

2 Supporting Figures

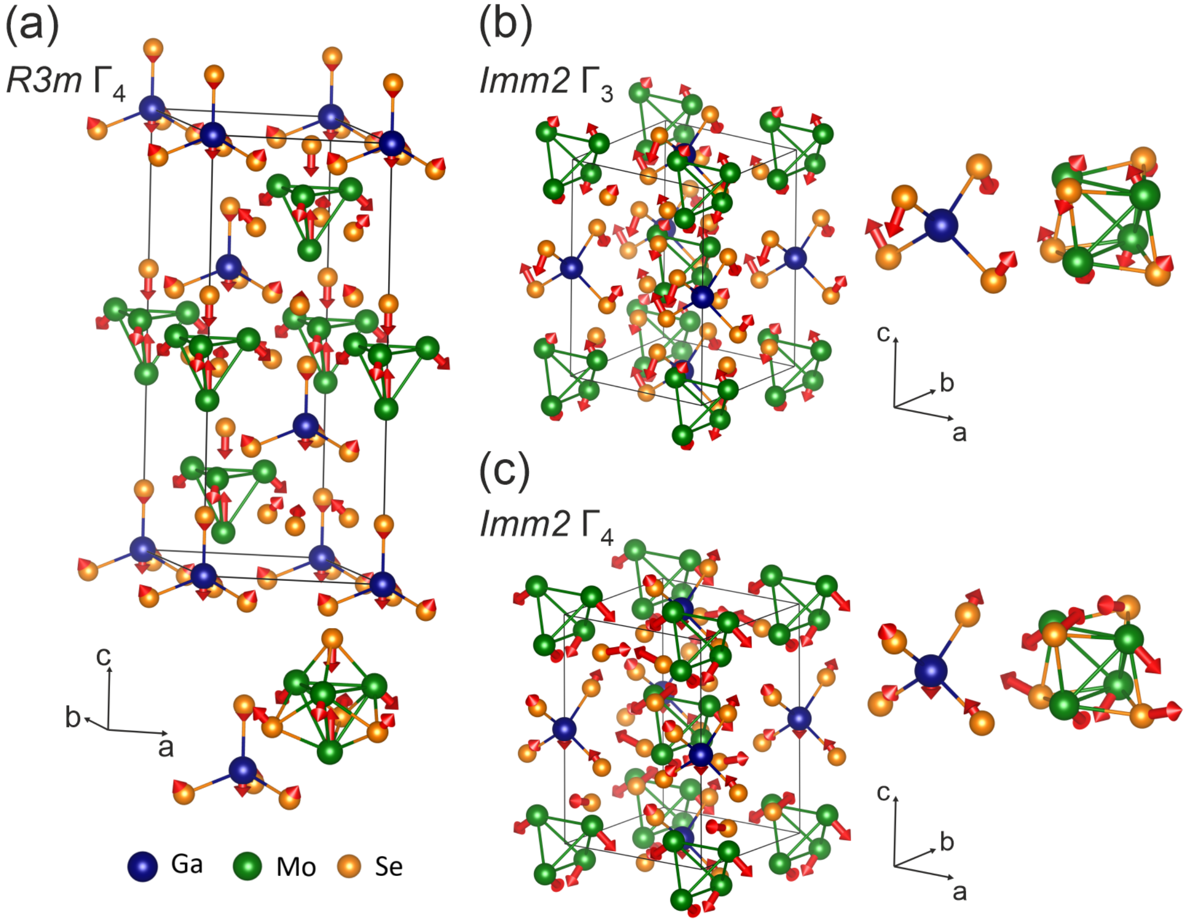

Figure S1: A representation of the effect on all atomic positions through the structural transition due to total effect of the (a) $R3m$ Γ_4 (b) $Imm2$ Γ_3 and (c) $Imm2$ Γ_4 atomic displacement modes. For each mode, both the full cell and the isolated Mo_4Se_4 and $GaSe_4$ units are visible. The axes of the $R3m$ (hexagonal setting) and $Imm2$ cells are shown. The length of the red arrows indicates the relative magnitude of the displacements between the room temperature and 2 K phases from the powder neutron diffraction. The figure was made using AMPLIMODES[3], STRCONVERT[4] and VESTA[5].

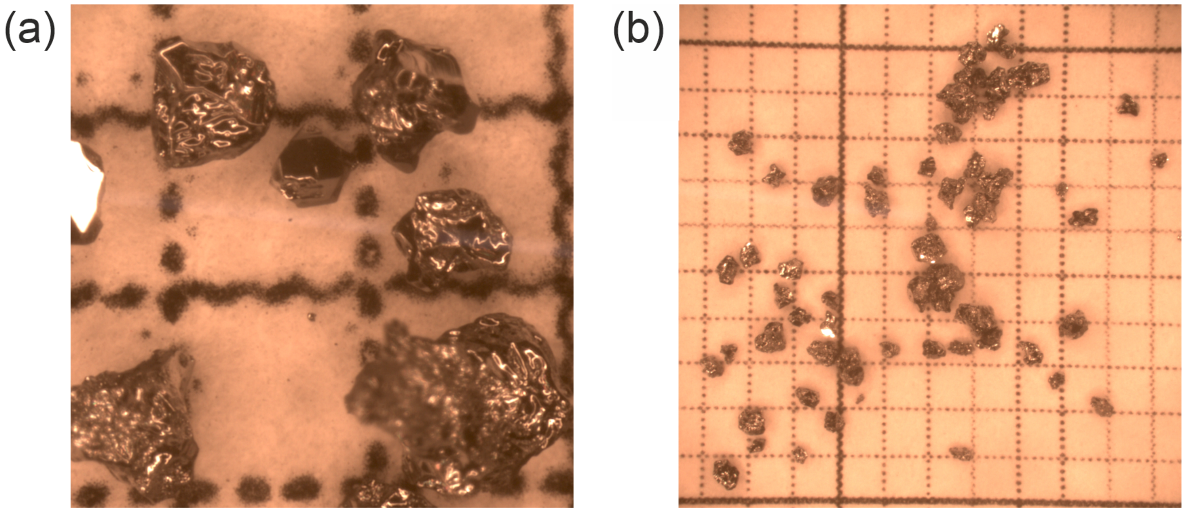

Figure S2: (a) A selection of crystals of $GaMo_4Se_8$ with rough edges, amongst which is a crystal with nicely defined facets. (b) The approximate quantity of crystals that can be extracted from a 2 g synthesis. Each square is 1 mm^2.

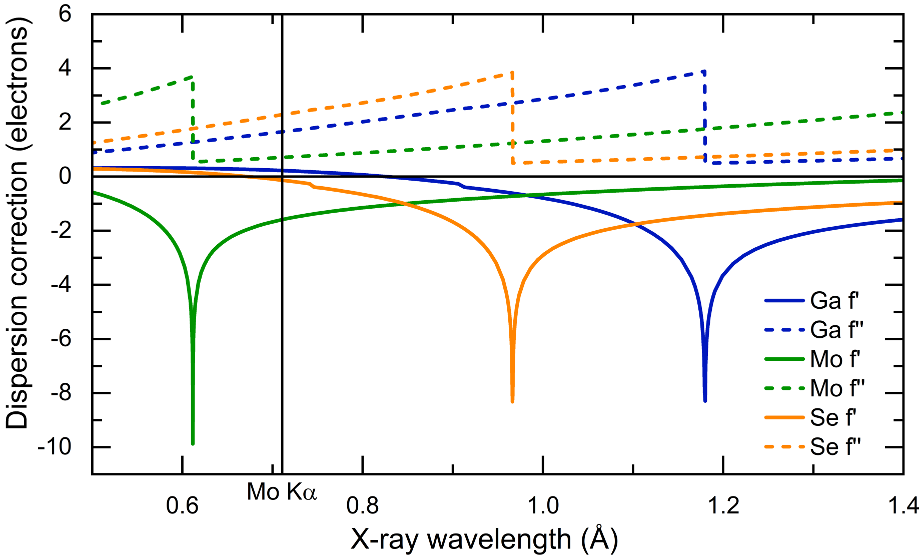

Figure S3: Calculated real (f') and imaginary (f'') anomalous scattering components for the elements Ga, Mo and Se as a function of X-ray wavelength.[6] The wavelength used in this SC XRD experiment (Mo K_α $\lambda = 0.71073$ Å) is indicated.

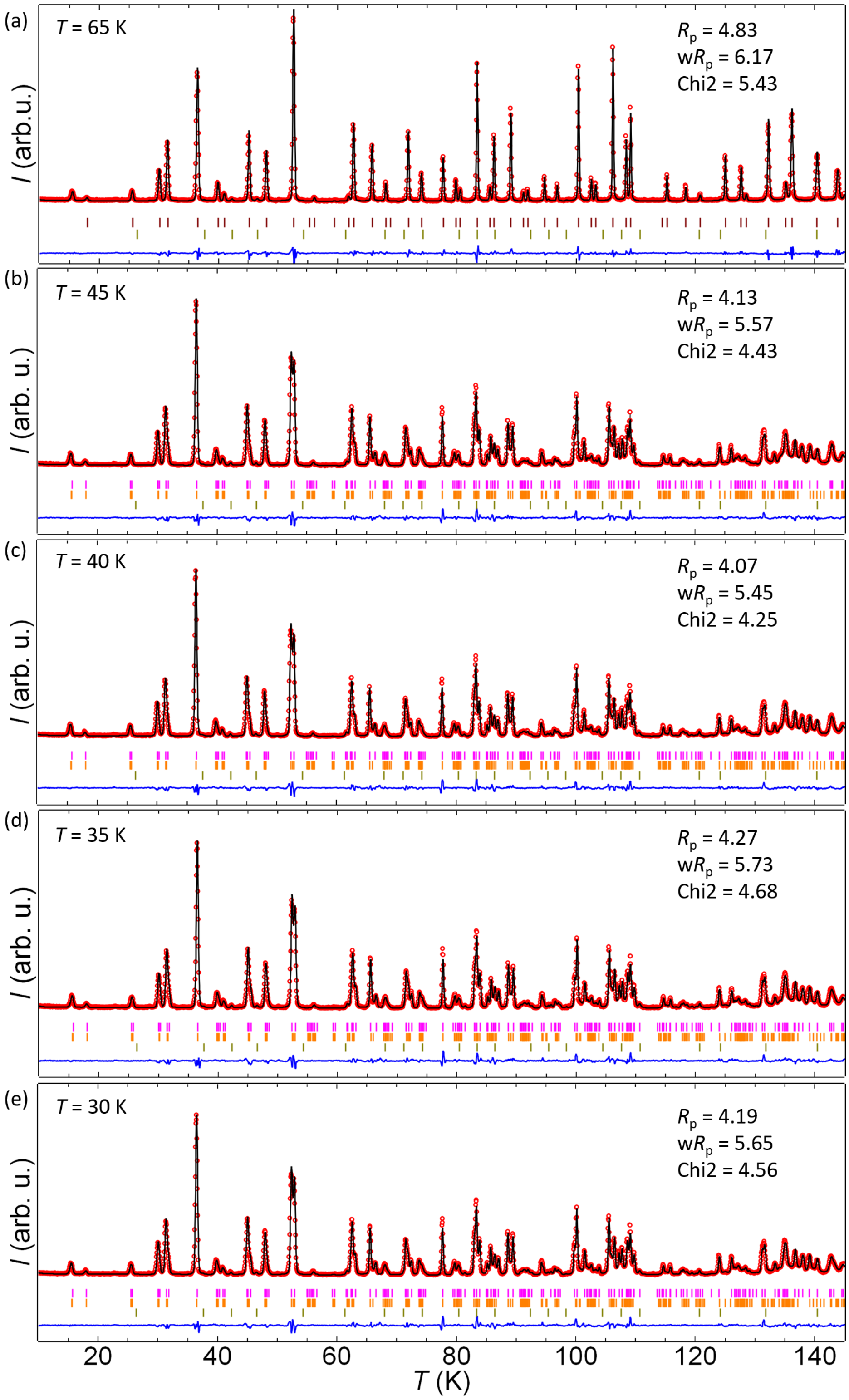

Figure S4: Experimental (red open circles) and theoretical (black line) powder neutron diffraction patterns along with the difference plot (blue line) measured at the temperatures (a) 65 K (b) 45 K (c) 40 K (d) 35 K and (e) 30 K. The R-factors of each refinement are presented inside the graph.

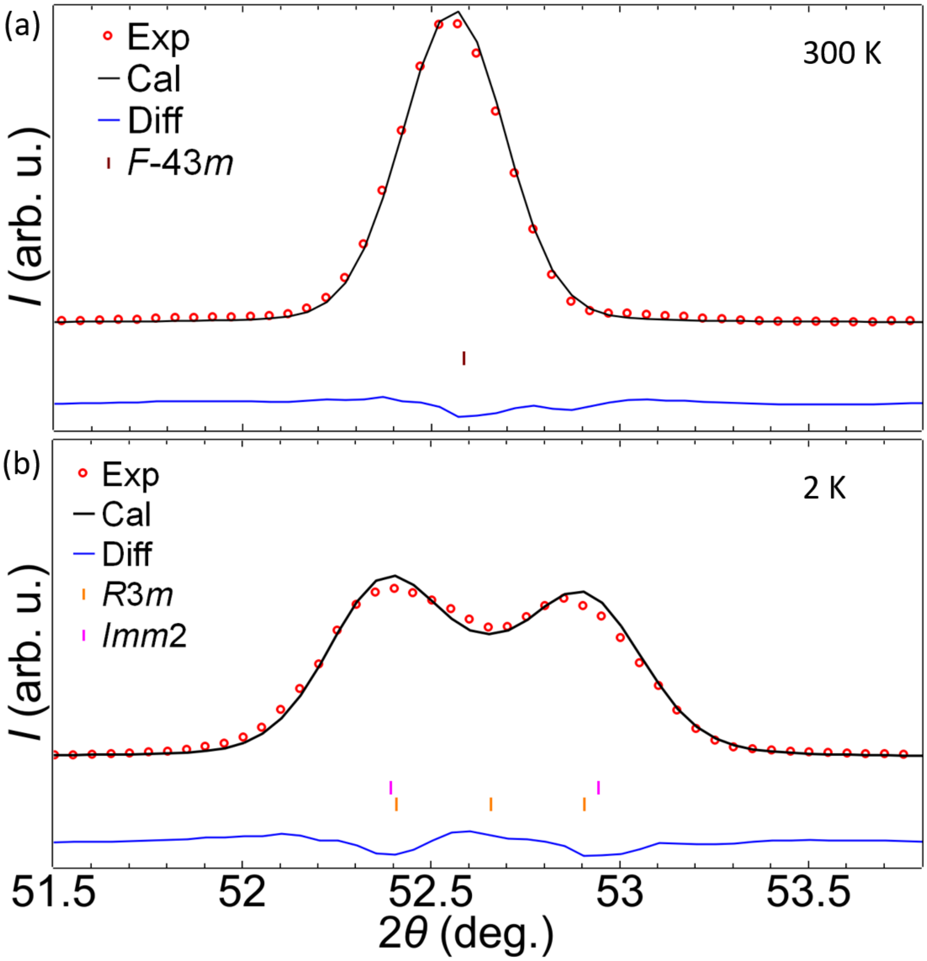

Figure S5: The most prominent peak (cubic 440) splitting in the powder neutron data, originating from the structural transformation.

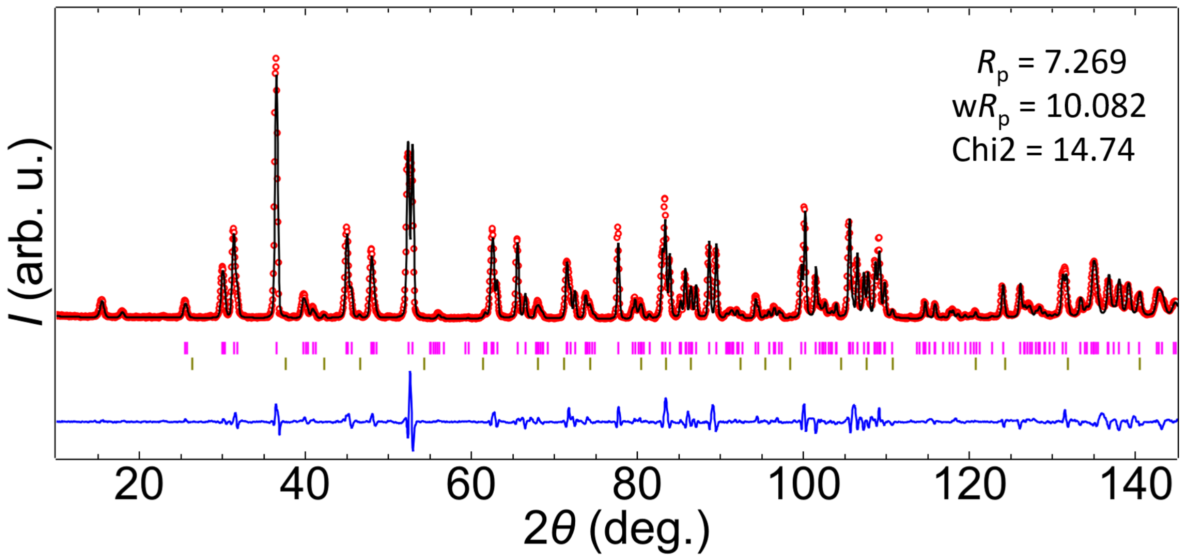

Figure S6: Experimental (red open circles) powder neutron diffraction data at 2 K and calculated (black line) data from a Rietveld refinement including only the $R3m$ phase, along with the difference plot (blue line).

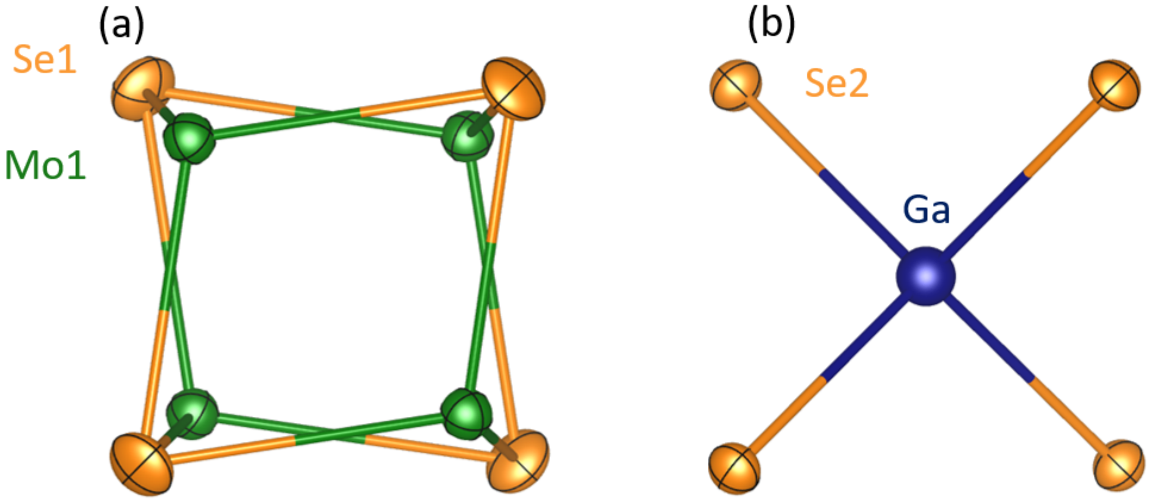

Figure S7: Displacement ellipsoids drawn at 99% probability level for the (a) $(Mo_4Se_4)^{5+}$ and (b) $(GaSe_4)^{5-}$ units from the room-temperature cubic phase powder neutron refinement.

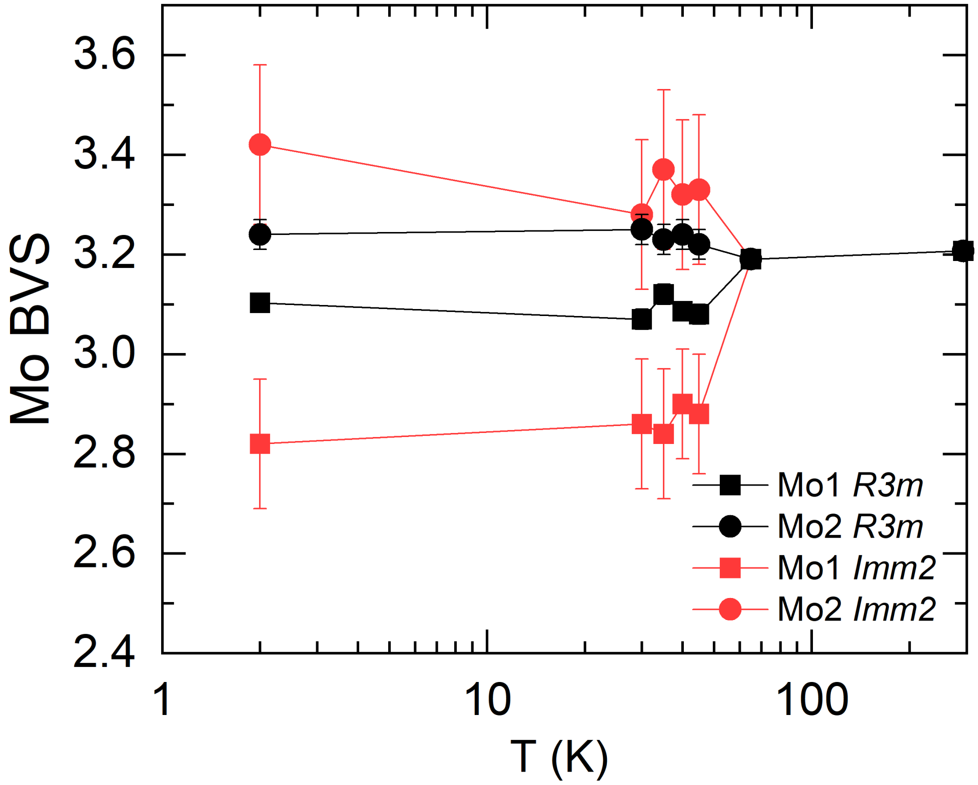

Figure S8: The approximate bond valence sum of the Mo ions in $GaMo_4Se_8$ as a function of temperature. The error bars for *Imm2* are much larger due to the larger errors in the atomic positions for that phase.

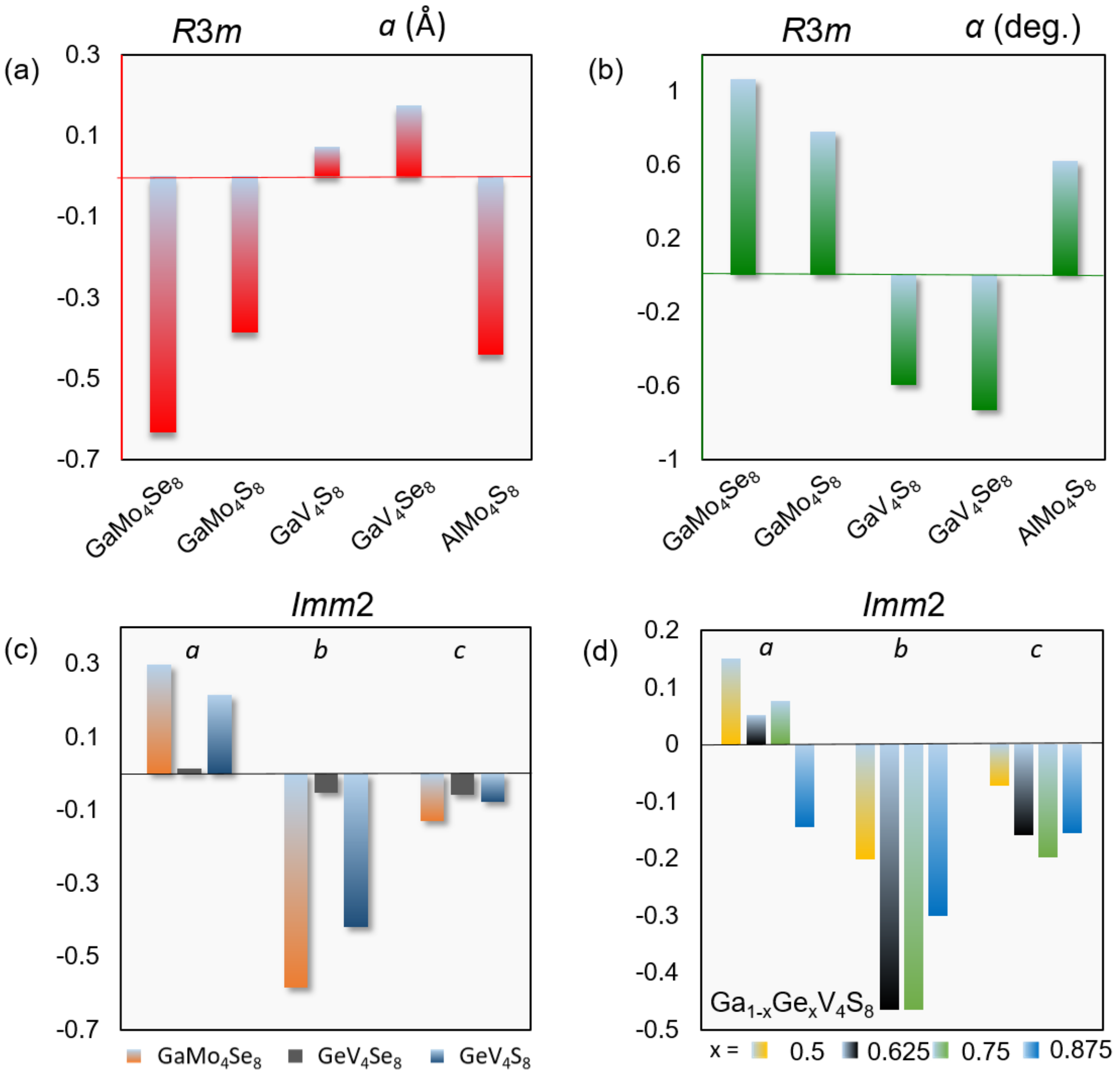

Figure S9: The percentage change in the cell parameters of the (a) a of R3m (b) rhombohedral angle α of $R3m$ (c) a, b and c of $Imm2$ in pure compounds (d) a, b and c of $Imm2$ in substitution compounds.[7–9]

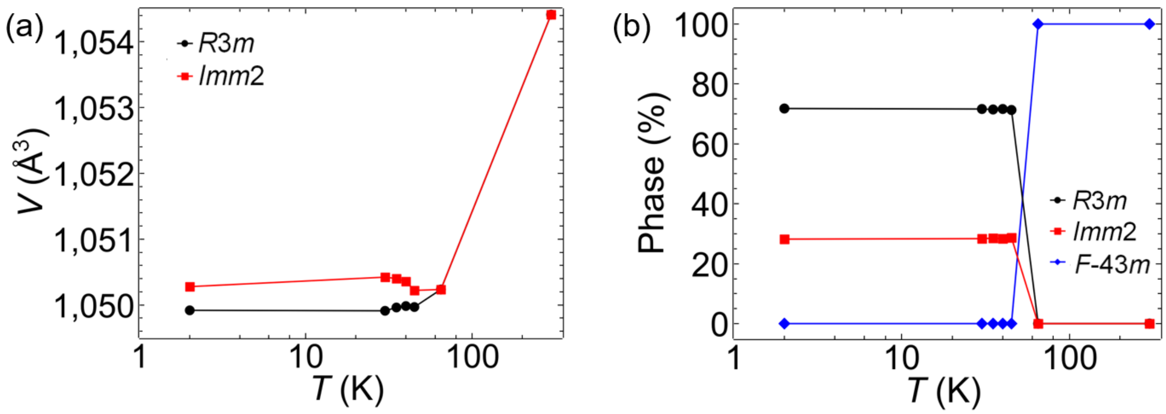

Figure S10: (a) The unit cell volume (b) phase percentage of R3m and Imm2 as a function of temperature.

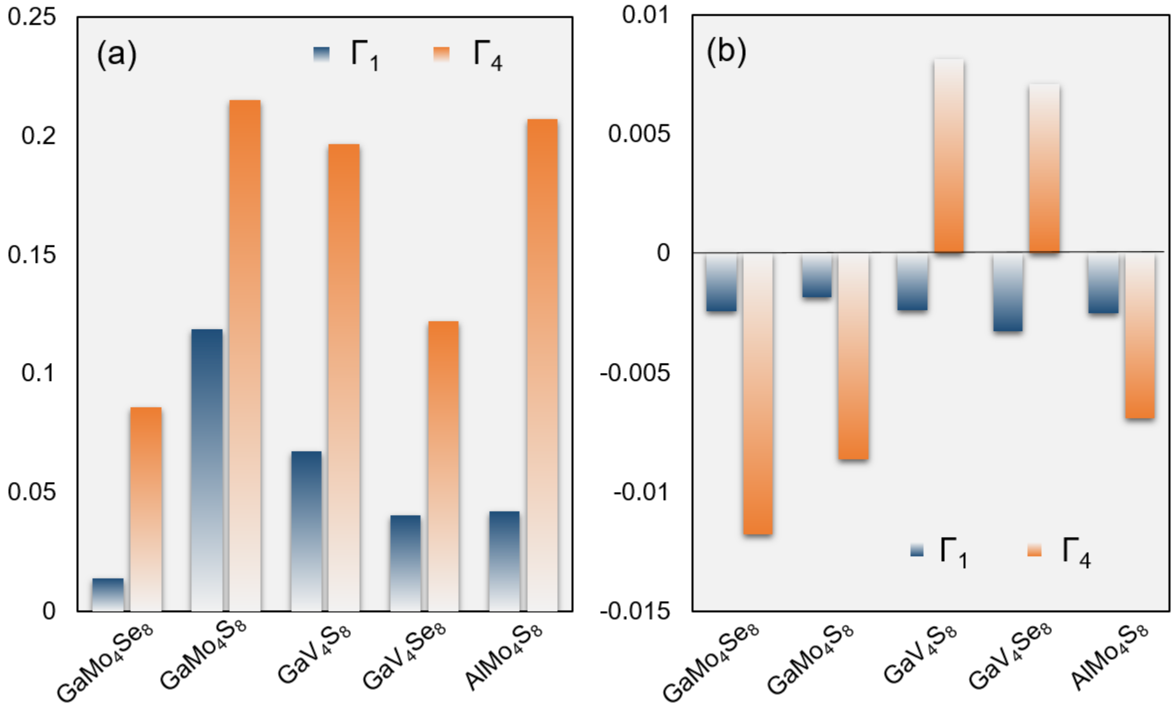

Figure S11: The distortion modes calculated for the $R3m$ phases of different lacunar spinel compounds. (a) Displacive modes and (b) strain modes.[7–9]

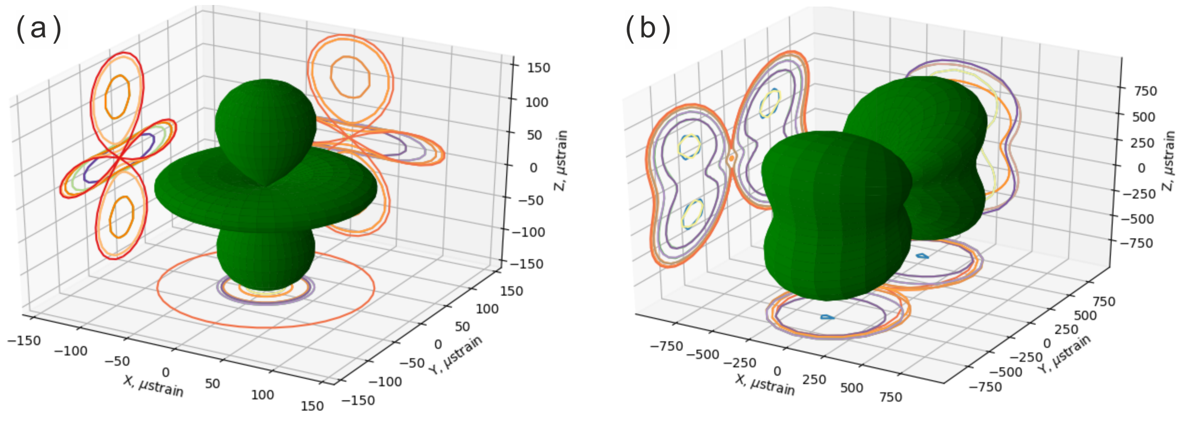

Figure S12: Anisotropic strain tensors from the 2 K PND data for (a) the $R3m$ phase and (b) the $Imm2$ phase, in cartesian coordinates. The axes are in arbitrary units as the values are scaled up from those in Table S4. Plotted in GSAS-II[10].

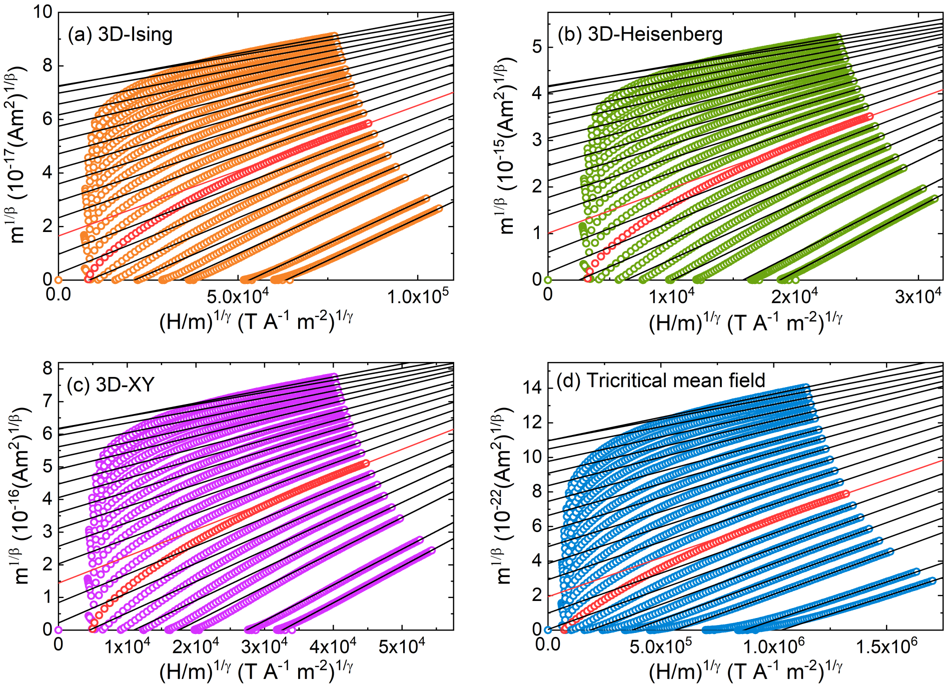

Figure S13: Arrott-Noakes plots using critical exponents from four theoretical models: (a) 3D-Ising model ($\beta = 0.325$, $\gamma = 1.24$), (b) 3D Heisenberg model ($\beta = 0.365$, $\gamma = 1.386$), (c) 3D-XY model ($\beta = 0.345$, $\gamma = 1.316$) and (d) tricritical mean-field model ($\beta = 0.25$, $\gamma = 1.00$). All linear lines have been fit to the high field data points (H = 5.6 T to 7 T) in each isotherm and extrapolated. The red data points and lines indicate the 27 K isotherm.

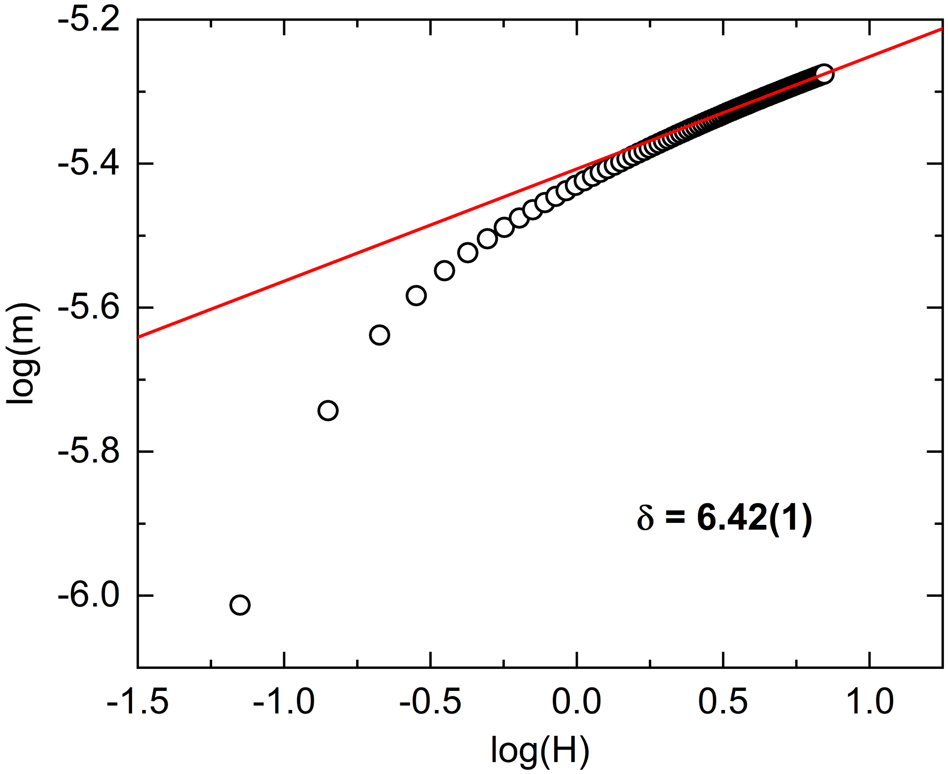

Figure S14: A plot of $log(m)$ vs $log(H)$ in the 27 K isotherm. The red line is a linear fit to the high field region (H = 5.6 T to 7 T), the gradient of which is used to calculate δ.

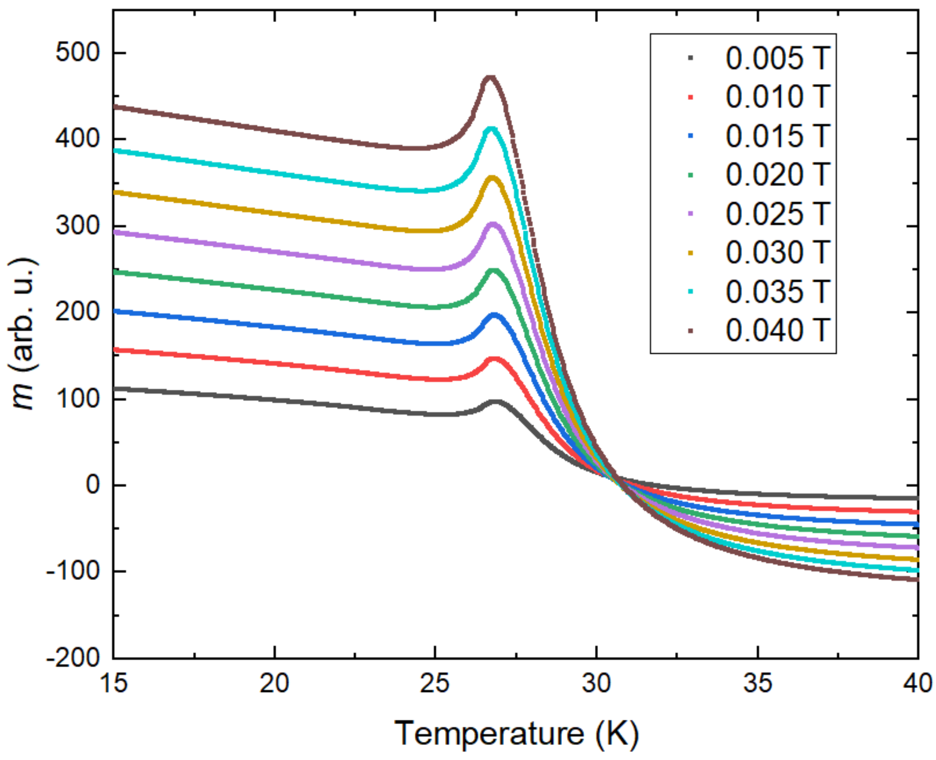

Figure S15: Scatter graphs of the raw data depicting the magnetisation of $GaMo_4Se_8$ against the corresponding temperature at which it was measured. Each line of the plot represents a specific field strength at the range of 0.005 to 0.040 T.

3 Supporting Tables

Table S1: Crystal 1 Fractional Atomic Coordinates and Isotropic Equivalent Atomic Displacement Parameters in $F\bar{4}3m$ (300 K) with $a = 10.2022(6)$ Å, $V = 1061.89(11)$ Å^3 and *wR(obs)* = 2.29, *R(obs)* = 1.86, *GOF(obs)* = 1.48 and Flack parameter = -0.06(6).

Label	Wyckoff	x	y	z	Occ.	U_{iso}
Mo1	16e	0.60083(8)	0.60083(8)	0.60083(8)	1	0.0025(2)
Se1	16e	0.36393(11)	0.36393(11)	0.36393(11)	1	0.0039(3)
Se2	16e	0.86354(11)	0.86354(11)	0.86354(11)	1	0.0031(3)
Ga	4a	0	0	0	1	0.0036(9)

Table S2: Crystal 3 Fractional Atomic Coordinates and Isotropic Equivalent Atomic Displacement Parameters in $F\bar{4}3m$ (100 K) with $a = 10.1654(6)$ Å, $V = 1050.45(11)$ Å^3 and *wR(obs)* = 1.51, *R(obs)* = 1.25, *GOF(obs)* = 1.15 and Flack parameter = -0.03(3).

Label	Wyckoff	x	y	z	Occ.	U_{iso}
Mo1	16e	0.39926(4)	0.39926(4)	0.39926(4)	1	0.00382(12)
Se1	16e	0.63631(6)	0.63631(6)	0.63631(6)	1	0.00465(18)
Se2	16e	0.13649(6)	0.13649(6)	0.13649(6)	1	0.00371(17)
Ga	4a	0	0	0	1	0.0048(4)

Table S3: Anisotropic displacement parameters (in Å^2) for $GaMo_4Se_8$ at 300 K with estimated standard deviations in parentheses. The anisotropic displacement factor exponent takes the form: $-2\pi^2[h^2a^{*2}U_{11} + \ldots + 2hka^*b^*U_{12}]$.

Label	U_{11}	U_{22}	U_{33}	U_{12}	U_{13}	U_{23}
Mo1	0.0033(2)	0.0033(2)	0.0033(2)	0.0001(2)	0.0001(2)	0.0001(2)
Se1	0.0050(4)	0.0050(4)	0.0050(4)	-0.0013(3)	-0.0013(3)	-0.0013(3)
Se2	0.0032(4)	0.0032(4)	0.0032(4)	-0.0003(3)	-0.0003(3)	-0.0003(3)
Ga1	0.0042(6)	0.0042(6)	0.0042(6)	0.00000	0.00000	0.00000

Table S4: The anisotropic microstrain tensor components resulting from the refinement of the 2 K PND data in FullProf for both $R3m$ and $Imm2$ phases.

Phase	S_{400}	S_{040}	S_{004}	S_{220}	S_{202}	S_{022}
R3m	0.042455	-	0.001671	-	-0.006305	-
Imm2	0.001180	1.733036	0.005011	0.160990	0.041944	1.384648

Table S5: The displacive mode amplitudes of each type of atom in the unit cell of the $R3m$ phase.

Mode	Amplitude (Å)
Γ_1[Mo1:e:dsp]A1(a)	-0.01252
Γ_1[Se1:e:dsp]A1(a)	-0.00564
Γ_1[Se2:e:dsp]A1(a)	0.00035
Γ_1 all	0.01374
Γ_4[Mo1:e:dsp]A1(a)	0.03281
Γ_4[Mo1:e:dsp]E(a)	0.05033
Γ_4[Se1:e:dsp]A1(a)	-0.04119
Γ_4[Se1:e:dsp]E(a)	-0.01818
Γ_4[Se2:e:dsp]A1(a)	-0.02531
Γ_4[Se2:e:dsp]E(a)	0.01615
Γ_4[Ga1:a:dsp]T2(a)	-0.02879
Γ_4 all	0.08583

Table S6: The displacive mode amplitudes of each type of atom in the unit cell of the $Imm2$ phase.

mode	Amplitude (Å)
Γ_1[Mo1:e:dsp]A1(a)	-0.01216
Γ_1[Se1:e:dsp]A1(a)	0.02121
Γ_1[Se2:e:dsp]A1(a)	0.05042
Γ_1 all	0.05603
Γ_3[Mo1:e:dsp]E(a)	0.11119
Γ_3[Se1:e:dsp]E(a)	0.01363
Γ_3[Se2:e:dsp]E(a)	0.07305
Γ_3 all	0.13374
Γ_4[Mo1:e:dsp]A1(a)	0.09496
Γ_4[Mo1:e:dsp]E(a)	0.06715
Γ_4[Se1:e:dsp]A1(a)	-0.02997
Γ_4[Se1:e:dsp]E(a)	-0.15931
Γ_4[Se2:e:dsp]A1(a)	0.06564
Γ_4[Se2:e:dsp]E(a)	-0.12349
Γ_4[Ga1:a:dsp]T2(a)	0.04127
Γ_4 all	0.24712

References

[1] Stephens, P. W. Phenomenological model of anisotropic peak broadening in powder diffraction. *J. Appl. Crystallogr.* **1999**, *32*, 281–289.

[2] Schueller, E. C.; Kitchaev, D. A.; Zuo, J. L.; Bocarsly, J. D.; Cooley, J. A.; Van der Ven, A.; Wilson, S. D.; Seshadri, R. Structural evolution and skyrmionic phase diagram of the lacunar spinel $GaMo_4Se_8$. *Phys. Rev. Mater.* **2020**, *4*, 064402.

[3] Orobengoa, D.; Capillas, C.; Aroyo, M. I.; Perez-Mato, J. M. AMPLIMODES: symmetry-mode analysis on the Bilbao Crystallographic Server. *J. Appl. Crystallogr.* **2009**, *42*, 820–833.

[4] Perez-Mato, J.; Gallego, S.; Tasci, E.; Elcoro, L.; de la Flor, G.; Aroyo, M. Symmetry-Based Computational Tools for Magnetic Crystallography. *Annu. Rev. Mater. Sci.* **2015**, *45*, 217–248.

[5] Momma, K.; Izumi, F. VESTA 3 for three-dimensional visualization of crystal, volumetric and morphology data. *J. Appl. Crystallogr.* **2011**, *44*, 1272–1276.

[6] Cromer, D. T.; Liberman, D. Relativistic Calculation of Anomalous Scattering Factors for X Rays. *J. Chem. Phys.* **1970**, *53*, 1891–1898.

[7] Powell, A. V.; McDowall, A.; Szkoda, I.; Knight, K. S.; Kennedy, B. J.; Vogt, T. Cation substitution in defect thiospinels: Structural and magnetic properties of $GaV_{4-x}Mo_xS_8$ ($0 \leq x \leq 4$). *Chem. Mater.* **2007**, *19*, 5035–5044.

[8] François, M.; Alexandrov, O. V.; Yvon, K.; Yaich-Aerrache, H. B.; Gougeon, P.; Potel, M.; Sergent, M. Structural phase transition in $GaMo_4Se_8$ and $AlMo_4S_8$ by X-ray powder diffraction. *Z. Kristallog.* **1992**, *200*, 47–55.

[9] Bichler, D. *Magnetismus und strukturelle Phasenumwandlungen von Verbindungen mit tetraedrischen Metallclustern*; Universität München, 2010; pp 75–83.

[10] Toby, B. H.; Dreele, R. B. V. GSAS-II: the genesis of a modern open-source all purpose crystallography software package. *J. Appl. Crystallogr.* **2013**, *46*, 544–549.
